# Unseen Care, Unequal Worlds: Photovoice Narratives of Siblings Supporting Deafblind Adolescents in Indonesia

**DOI:** 10.12688/f1000research.171949.1

**Published:** 2026-01-19

**Authors:** Hastin Trustisari, Fentiny Nugroho, Dini Widinarsih, Richard Mottershead, Muhammad Arsyad Subu

**Affiliations:** 1 Department of Social Welfare, Faculty of Social and Political Sciences, University of Indonesia, Depok, Indonesia; 2 Department of Social Welfare, Faculty of Business and Social Science, Universitas Binawan, Jakarta, Indonesia; 3 SEHA, Sakina, Behavioural Science Institute, Al Ain, United Arab Emirates; 4 College of Nursing, University of Baghdad, Baghdad, Iraq; 5 Department of Nursing, College of Health Sciences, University of Sharjah, Sharjah, United Arab Emirates; 6 Faculty of Nursing and Midwifery, Universitas Binawan, Jakarta, Indonesia

**Keywords:** Deafblindness; siblings; photovoice; transition; social work; Indonesia.

## Abstract

**Background:**

Deafblindness is a rare and complex condition that is often accompanied by additional intellectual and mental disabilities. Research on adolescents with deafblindness during the post-school transition remains limited, with existing studies primarily focusing on parental perspectives. Sibling voices, particularly in Indonesia, where services are scarce and family-based care is the dominant approach, are underexplored.

**Aim:**

This study examined how siblings in rural and urban Indonesia manage caregiving and roles during the transition of adolescents with deaf-blindness from school to family.

**Methods:**

Using a participatory Photovoice approach, eight siblings (aged 15–25) documented their daily realities through photographs, semi-structured interviews, and focus group discussions over a 12-week period. Data were thematically analyzed.

**Results:**

Six themes emerged: invisible care work, emotional strain, rural–urban divides, social relationships and stigma, temporal transitions, and bridging advocacy. Urban siblings reported tension between education, employment, and caregiving, whereas rural siblings encountered stigma and limited services, but experienced stronger kinship support. Across various contexts, siblings often act as hidden caregivers and advocates, yet their contributions remain undervalued.

**Conclusions:**

Supporting siblings requires addressing the intersecting dynamics of geographic area, culture, and disability care. Social work professionals must recognize both their challenges and their resilience, which helps them balance caregiving and personal development in the transition phase. Longitudinal research is needed to capture evolving sibling roles across the deafblind transition landscape.

## Introduction

Deafblindness is a rare condition that often occurs alongside multiple disabilities of varying severity, yet it is frequently overlooked. ^1, 2^ Deafblindness is a condition characterized by different levels of varying degrees of hearing and vision loss, which affects communication and the ability to obtain information. It can result from congenital factors, age-related changes, genetics, prenatal infections, and complications. ^3, 4^ This can impact a person’s independence and necessitate assistance throughout their life. ^3, 5, 6^

Deafblindness is often considered axiomatic, a minority, the most vulnerable group, and invisible to the general public. ^7– 9^ This reality often causes them to be excluded from development programs, marginalized in policy planning, and even in the disability program itself. ^2, 9^ Additionally, about 0.2% of the world’s population lives with severe deafness and blindness, while “milder forms” of deafness and blindness impact about 2% of the global population. ^10^

The existence of people with deafblindness complexity can have emotional consequences, lifelong parenting needs, and a large financial burden on the family. ^11^ Socially, the presence of individuals with deafblindness poses challenges in building interactions and becomes a burden on other family members ^12– 14^ Especially deafblind teenagers who are facing a transition period. The transition focuses not only on the shift from school to adult life but also encompasses aspects of independent living, social participation, and access to community services. ^15, 16^

At this stage, the bleak picture is often characterized by job uncertainty, difficulties in living adulthood, decreased life skills and independence, as well as health. ^17– 21^ Findings even reinforce the fact that individuals with deafblindness, very few live independently post-school and require family roles past this stage. Ultimately, family members play a fundamental role as the primary and first source of support for individuals with deafblindness. ^22, 23^ Family plays a crucial role because individuals with deafblindness often face limited independent living due to low service and policy support. ^13, 21, 24, 25^ Most research on deafblind families has been conducted in developed countries, which tend to have relatively stronger social service support.

However, in the context of developing countries, the experience of siblings with multiple disabilities, especially the deafblind, can differ significantly. In Indonesia, the culture of parenting is often deeply rooted in multigenerational households, reflecting family values that encompass both the nuclear and extended families. Although it has begun to shift, family-centered parenting is not easy to abandon. Access to education and health services is still unequal between cities and villages, while social stigma against disabilities and siblings also remains strong. Siblings experience communication challenges, cultural gaps, kinship, and spirituality that are still challenges in Indonesia. ^26– 28^

For this reason, it is necessary to find ways to understand siblings’ perspectives on the issue of deafblindness transition by considering local culture. ^6, 14, 15, 29, 30^ However, unfortunately, studies on deafblind individuals and their families are still few ^11^ In fact, the fulfillment of the quality of life of the deafblind is influenced by the right interaction, support, and connection with the family ^8, 31^

Use of photovoice to research the roles and experiences of siblings living with adolescent deafblindness during the transition phase has been rarely explored. Considering that the majority of photovoice research focuses more on the experiences of people with disabilities themselves. ^32– 37^ Although studies have highlighted the impact of siblings, they are limited to specific types of disabilities and do not focus on the transition phase. ^38^ This method is thought to allow siblings to express their voices in a definitive way through visuals and narratives, while opening up space to explore experiences hidden in formal spaces. ^39^

Research focusing on the voices of siblings with deafblindness during the transition period was very limited. The perspective of siblings is still underrepresented in disability studies, as parental voices have historically dominated. ^11, 40^ This fact results in sibling experiences often being ignored and receiving less attention. ^40, 41^ recommended that for the study of adolescent deafblindness, it is necessary to include siblings in different regions according to the geographical context. The absence of geographically based research involving diverse cultures and economies in which siblings play a role is a strong reason why this research is important, as it provides diverse and inclusive perspectives.

### Research objectives

This study aims to describe the experiences and roles of siblings who live with deafblind adolescents, particularly those who face mental and intellectual barriers during the family transition phase.

### Research questions

How do sibling experiences and roles support deafblind adolescents who face mental and intellectual challenges during their transition in Indonesia?

## Method

### Design

This study used photovoice, which is a flexible tool that can be adapted for specific participatory purposes and is used with diverse groups facing various issues. ^42^

Thus, it enables siblings of deafblind adolescents with mental and intellectual disabilities to document their experiences and roles during the transition from school to home, and to express their voices in a definitive way through visuals and narratives, while opening up space to explore experiences hidden in formal spaces. ^39^

Previous studies have highlighted the impact of siblings, but have been limited to specific types of disabilities and have not focused on the transition phase. ^38^ It has mostly focused on the experiences of people with disabilities themselves. ^32– 36^

The three main objectives of Photovoice are: (1) to provide opportunities for people to record and reflect on the strengths and concerns of their communities, (2) to encourage critical dialogue and knowledge on important issues through photographs, and (3) to reach out to policymakers on issues. ^42^

Currently, Photovoice is considered a creative research approach that combines photography with qualitative methods by involving the active participation of participants. ^33, 43^ Although photovoice etymologically implies visual and verbal communication, this method enables participants to express their world visually, thereby opening up a space for their perspectives to be heard, because often experience cannot be captured through conventional methods. ^33, 44^ The use of photovoice has also been shown to be a meaningful strategy for communities to raise awareness about health and social issues. ^45^

Data collection in this study involved nine steps of photovoice combined with semi-structured interview procedures and focus group discussions. ^46– 48^ This method was chosen to strengthen the perspective of adolescents with deafblindness who live in different geographic areas and are often overlooked in disability research. Thematic analysis was employed to identify patterns, with a focus on the dynamics of experiences and the role of sibling involvement in rural and urban areas of Indonesia. This is to ensure that siblings’ voices and experiences are recognized as valuable knowledge for understanding the dynamics of brotherhood in families with individuals who are deafblind and have mental and intellectual disabilities.

### Setting and participants

This research was conducted in both rural and urban areas of Indonesia, where family-based parenting practices are deeply ingrained in local culture. Eight participants were siblings of deafblind adolescents with mental and intellectual disabilities ( Table 1 and Table 2), selected purposively through special education schools, and assisted by teachers and social workers, to ensure the suitability of the characteristics that have been determined. The participant criteria were siblings of either boys or girls aged 15–25 years ( Tables 3 and 4) who lived together and provided support to deafblind adolescents during the transition period. Written informed consent was obtained from all participants aged 18 years and older. For participants under 18, written parental consent was obtained from parents or legal guardians, and verbal assent was obtained from the adolescents themselves after the study purpose and procedures were clearly explained in accessible language. This study did not recruit participants with communication disorders or severe cognitive limitations due to potential barriers to meaningful participation in the photovoice activities.

**Table 1.** Deafblind characteristics in urban areas.

Initial name	Age (y.o)	Gender	Degree of hearing loss	Degree of vision loss	Additional disabilities	Communication mode	Educational placement
D1	18	Female	Profound	profound	Intellectual disability, epilepsy	Sign language, tactile	Non-government school
D2	19	Female	Moderate	Profound	Intellectual disability, autism	Sign language, tactile	Non-government school
D3	20	Female	Severe s	Severe	Intellectual dan mental disability	Sign language, tactile	Non-government school
D4	19	Male	severe	Moderate	Mental disability, cerebral palsy	Sign language, tactile	Non-government school

Table shows the characteristics of deafblind adolescents living in urban areas and attending private or non-government schools in Indonesia.

**Table 2.** Deafblind characteristics in rural areas.

Initial name	Age (y.o)	Gender	Degree of hearing loss	Degree of vision loss	Additional disabilities	Communication mode	Educational placement
A1	17	Male	Profound	Profound	Intellectual disability,	Sign language, tactile	Government school
A2	20	Male	severe	Profound	Cerebral palsy, intellectual disability	Sign language, tactile	Government school
A3	19	Female	Severe	Severe	Intellectual disability	Sign language, tactile	Government school
A4	19	Male	Moderate	Moderate	Mental dan intellectual disability,	Sign language, tactile	Government school

Table shows characteristics of deafblind adolescents living in rural areas who attend public or government schools in Indonesia.

**Table 3.** Sibling demographic background in urban areas.

Participant	Age	Gender	Position in the family	Family context	Parents’ marital status	Socioeconomic status
1	20	Female	Older sister	Nuclear family	Married	Higher
2	19	Male	Younger brother	Nuclear family	Divorce	Lower
3	25	Female	Older brother	Extended family	Single parent	Higher
4	21	Female	Older sister	Nuclear family	Married	Middle

Table shows the background of siblings/participants living in urban areas in Indonesia.

**Table 4.** Sibling demographic background in rural areas.

Participant	Age	Gender	Position in the family	Family context	Parents’ marital status	Socioeconomic status
5	16	Female	Younger sister	Nuclear family	Married	Higher
6	17	Male	Younger brother	Nuclear family	Married	Lower
7	25	Female	Older sister	Extended family	Divorce	Higher
8	23	Female	Older sister	Extended family	Single parent	Middle

Table shows the demographic background of siblings/participants living in rural areas in Indonesia.

### Data collection

This study combines the 9 steps of photovoice as summarized in Table 5. ^33, 42, 46^ The following is an explanation of each step of the photovoice procedure applied by the researcher:

**Table 5.** Photovoice timeline (9 Steps, 12 Weeks).

Step	Period	Description
Step 1	Week 1	Selection stage
Step 2	Week 2	Recruit participants, build contacts, and make introductions
Step 3	Week 3	Ethical photovoice training
Step 4	Week 4-5	Photo Capture and Visual Data Production
Step 5	Week 6	Photo-elicitation interviews, Categorization of themes
Step 6	Week 7	Group discussion with SHOWeD, Personal narrative & photo meaning
Step 7	Week 8-9	Co-analysis of photos & narratives, Grouping categories/themes, and critical analysis
Step 8	Week 10-11	Photo exhibitions in schools/communities, Sharing sessions with stakeholders, Documentation & publication of results
Step 9	Week 12	Evaluation of participation experience, action plan, and recommendations

Table shows the 9-step photovoice resume applied to the study.

### Photovoice procedure

The data collection lasted 12 weeks and consisted of 9 structured steps. Each phase is described in detail below to ensure reproducibility.

1. Preparation (Weeks 1–2).
  The researcher contacted the school and social worker, then explained the purpose of the study. After that, parents of siblings under 18 years old, and all siblings, are invited to receive an explanation of the research process and ethical principles. Furthermore, the researcher obtained written consent from parents and siblings.
2. Ethical Photography basic training (Week 3).
  Each participant attended a 90-minute face-to-face training session. Training includes using cameras, obtaining consent to take photos, protecting privacy, and avoiding identifiable faces unless permission is obtained.
3. Equipment and Appliances
  Each participant is free to use their camera or smartphone device. A standard DSLR camera with 12-24 MP or higher, or a smartphone with 64 MP or higher, is recommended. Digital audio recorders are used for interviews and FGDs. All data is stored on a password-protected device.
4. Photo Collection (Weeks 4–5).
  Participants were asked to take 10–15 photos each week depicting their parenting experiences, emotions, and daily challenges. The researcher communicated with participants through WhatsApp groups to ensure consistency and answer questions.
5. Photo Interview collection (Week 6).
  Individual interviews were conducted at the participant’s home or school, lasting 45–60 minutes. Each participant chose 5–7 meaningful photos and explained their story using the SHOWeD framework ("What Do You See Here? What Happened? How does this relate to our lives? Why does this situation exist? What Can We Do?").
6. Focus Group Discussions (Weeks 7–9).
  Two FGDs (one for urban participants and one for rural participants) were held in the school’s meeting room, each lasting 90 minutes. The discussion was moderated by the author and observed by a research colleague who made reflective field notes.
7. Joint Analysis and Thematic Grouping (Weeks 8–9).
  Participants collaboratively grouped their photos and narratives into categories that represented shared experiences and roles. The researcher facilitates reflection to develop the themes that emerge.
8. Community Exhibition and Reflection (Week 10-11).
  The participants chose photos to display on the information board pasted on the school wall. This step encourages public awareness and internal and external policy dialogue in schools.
9. Evaluation and Action Planning (Week 12).
  The closing workshop allowed participants to reflect on the Photovoice process, discuss the learnings gained, and propose a plan for future advocacy. In total, the participants produced 47 photos, of which 18 were selected from 4 interviews and 2 FGDs, forming a primary data set for analysis.

### Data analysis

**Figure 1.**
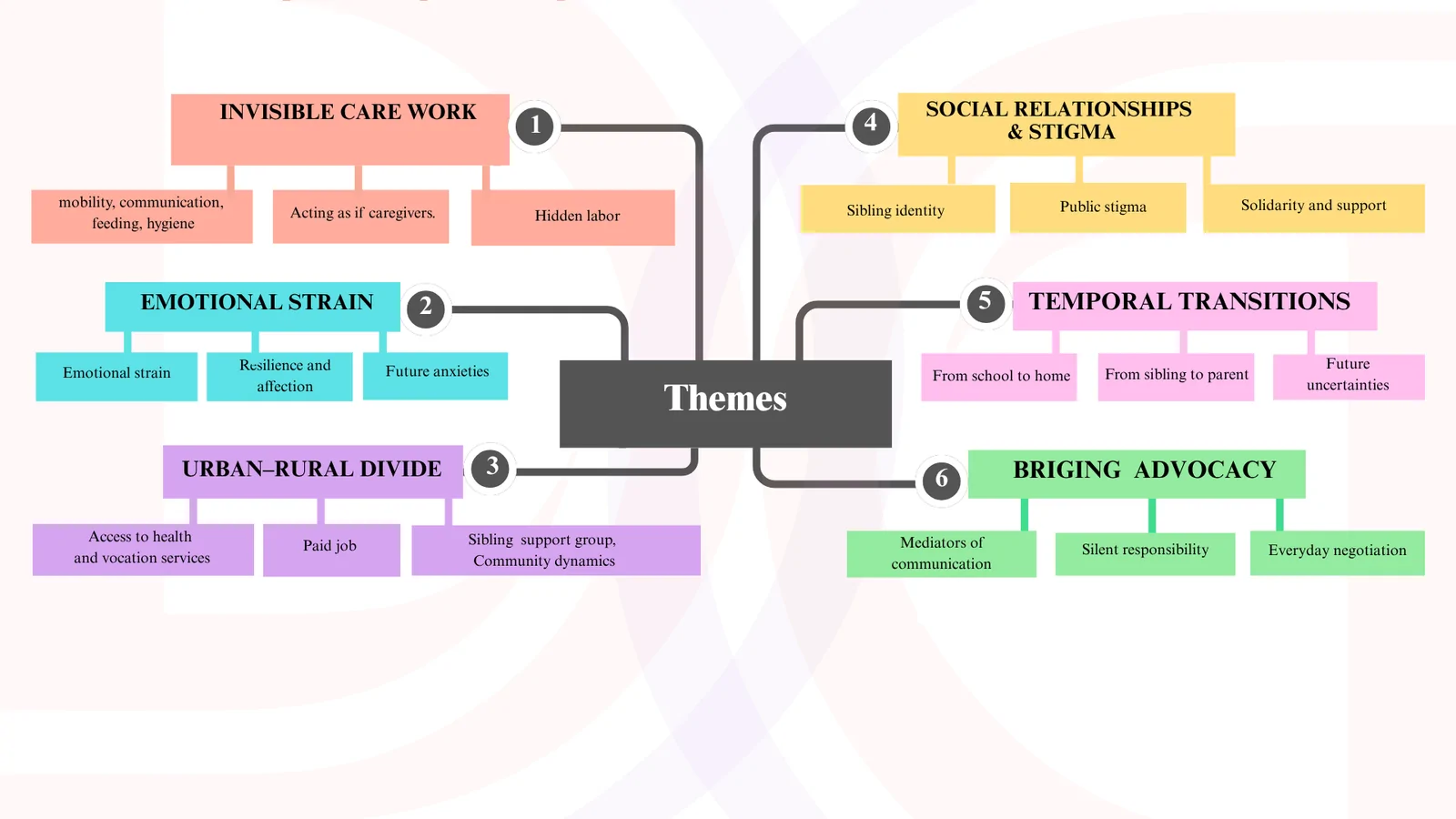
Thematic analysis results.

This Photovoice study adapted thematic analysis involves six systematic stages ^49^ which raises themes as visualized in Figure 1. The first stage involved re-transcribing the interview and discussion, and examining the photos produced by the participants to capture the initial meaning. The second stage involves generating initial codes, which entail coding the participant’s narrative and the photo’s visual elements, including both descriptive and interpretive codes. The third stage, the search for themes stage, involves grouping codes into broader patterns to form an initial theme that represents the siblings’ experience and their role as participants.

Furthermore, the fourth stage, reviewing themes, involves assessing the suitability of the theme in relation to the overall data and photos to ensure the accurate representation of the siblings’ experience and role. Fifth, themes are defined and named, which involves specifying the boundaries and essence of each theme while assigning meaningful names to the photos. In the final stage of producing the report, the researcher compiles a reflective and analytical report by integrating photos, narratives, and direct quotes to reinforce the theme that spans two study locations: urban and rural.

### Ethical consideration and consent

Ethical approval was obtained from the Research Ethics Committee of the Department of Social Welfare Sciences, Faculty of Social and Political Sciences, University of Indonesia (No. S-845/UN2. F9. D1/PPM. 00.04/2023) dated December 13, 2023. Participants and parents provided written consent for participation and the use of anonymized photos. Data were anonymized, and all personal identities were removed prior to publication. Additionally, we obtained written consent from all study participants or their parents to publish their details.

In the initial step, all participants were informed about the objectives and the phasing of the photovoice approach in this study. We also provide training on ethical procedures for documenting research, including the use of images and photos, to ensure compliance with research ethics standards. The study utilized audio recordings to facilitate the transcription of interview and group discussion results. We also provide a dedicated room for interview sessions and group discussions, which facilitates photo categorization while ensuring confidentiality and maintaining consistency.

### Rigor

In this study, we applied the criteria of credibility, dependability, confirmability, and transferability. ^50^ Triangulation of data sources, including photo documentation, interviews, and group discussions involving teachers, parents, and social workers, was used to increase credibility. Discussions between participants and researchers reinforce dependability, while participant feedback on themes and the resulting initial categorization are used to improve confirmability. An in-depth description of the participant’s context and geography is provided to support the transferability of the findings.

## Result

### Theme 1. Invisible care work

This theme reflects on the burden of care and assistance carried out by siblings in both urban and rural families, which includes 3 sub-themes as follows:

***Subtheme: mobility, communication, feeding, hygiene***

**Figure 2.**
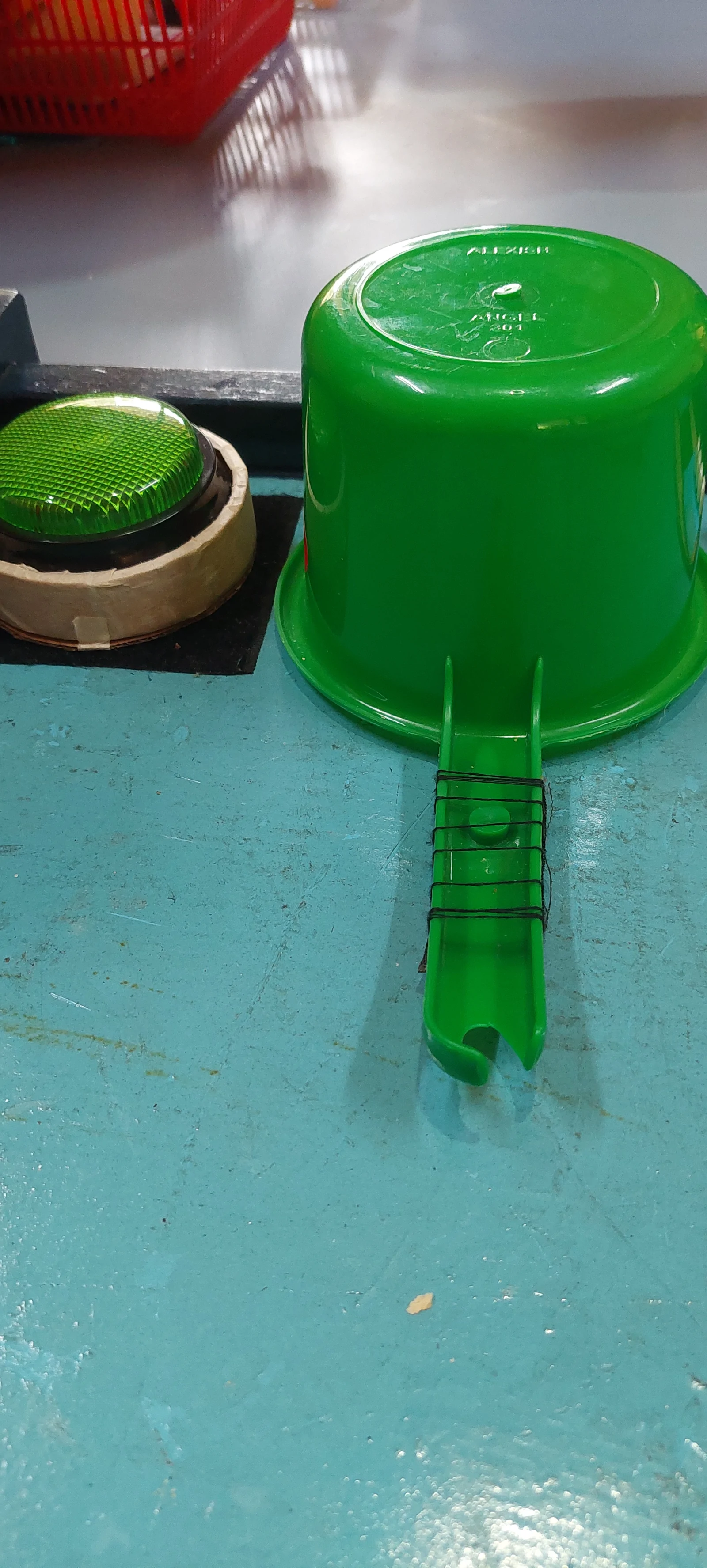
A dipper and sound button.

Male and female siblings reported that during the transition period, they accompanied them to maintain daily personal hygiene, such as bathing, feeding, and assisting with toileting activities. For siblings in the city, this activity is often considered very time-consuming and can test patience, especially in small families without neighborly support. Meanwhile, in the village context, they are occasionally assisted by relatives living nearby, though this assistance is inconsistent. Figure 2 shows the dipper symbol and the sound button (a dipper is a bath tool used in Indonesia).

The urban sibling admitted that the school had equipped an augmentative alternative communication (AAC) tool in the form of a voice button. Deafblind adolescents are taught to use it when they want to use the toilet. But the fact is that it is not consistently done at home. Some daily activities can lead to family conflicts when people fail to communicate effectively, resulting in misunderstandings. Siblings spend a lot of time tidying up, sweeping, cleaning up leftovers, avoiding tantrums, and accompanying their parents on walks. Although they also admit that when they have free time, they often leave it unstructured because they are unsure what to do.

“… *I don’t understand how to help my brother, because often he suddenly throws tantrums and hits when I’m in company. I can’t understand what he wants. Because I don’t understand how to communicate with him*…” *(Participant 3).*

***Subtheme: Caregiver role***

All siblings admit that most of their deafblind siblings’ activities require help and close supervision. The task of tidying up rooms is tiring and never seems to end.

*“When my sister entered the room, she started to ruffle the contents of the closet. I don’t know what I was looking for, but after I tidied it up, it was still dismantled. This is what sometimes makes me impatient and feel tired,” (Participant 8).*

***Subtheme: Hidden worker***

Siblings in both rural and urban areas report doing domestic work regularly. An activity that appears simple but actually reveals the hidden reality of how siblings serve as unseen workers. They play a significant role in preparing food, supervising household chores, and maintaining cleanliness.

*“…Honestly, I feel like I’ve lost my playing time, because of the non-stop but invisible domestic work*… *” (Participant, 5).*

The identities of “brother” and “domestic worker” are often conflated, creating emotional burdens, exhaustion, and frustration.

### Theme 2: Emotional stain

The second theme is emotional strain, which reflects the emotional dynamics experienced by siblings when supporting adolescents with deafblindness, which includes 2 sub-themes, namely resilience and affection: future and anxiety. Siblings often feel torn between affection, resilience, and concern for the long-term future. Almost all siblings reported experiencing emotional tension during the transition period of deafblind adolescents. Deafblind adolescents cannot be taken out alone to use public transportation or allowed to interact with strangers. There is a mixture of love and hidden tiredness because they must ensure safety and comfort around familiar people.

*“…It’s often called strange, because all the work has to be accompanied, where to go and not want to be helped by strangers, very tired but yes very dear.” (Participant 4).*

***Subtheme: Resilience and affection***

Emotional stress often conflicts with personal life, expectations, care responsibilities, and affection. Siblings in urban areas recognize that urban environments offer more opportunities in academics and employment, as well as public and social facilities, but require greater resilience.

*"Leaving and returning from work, I faced an extraordinary traffic jam. Arriving home, I couldn’t rest because I still accompanied my sister, alternating with my mother. I don’t even have time alone" (Participant 3).*

**Figure 3.**
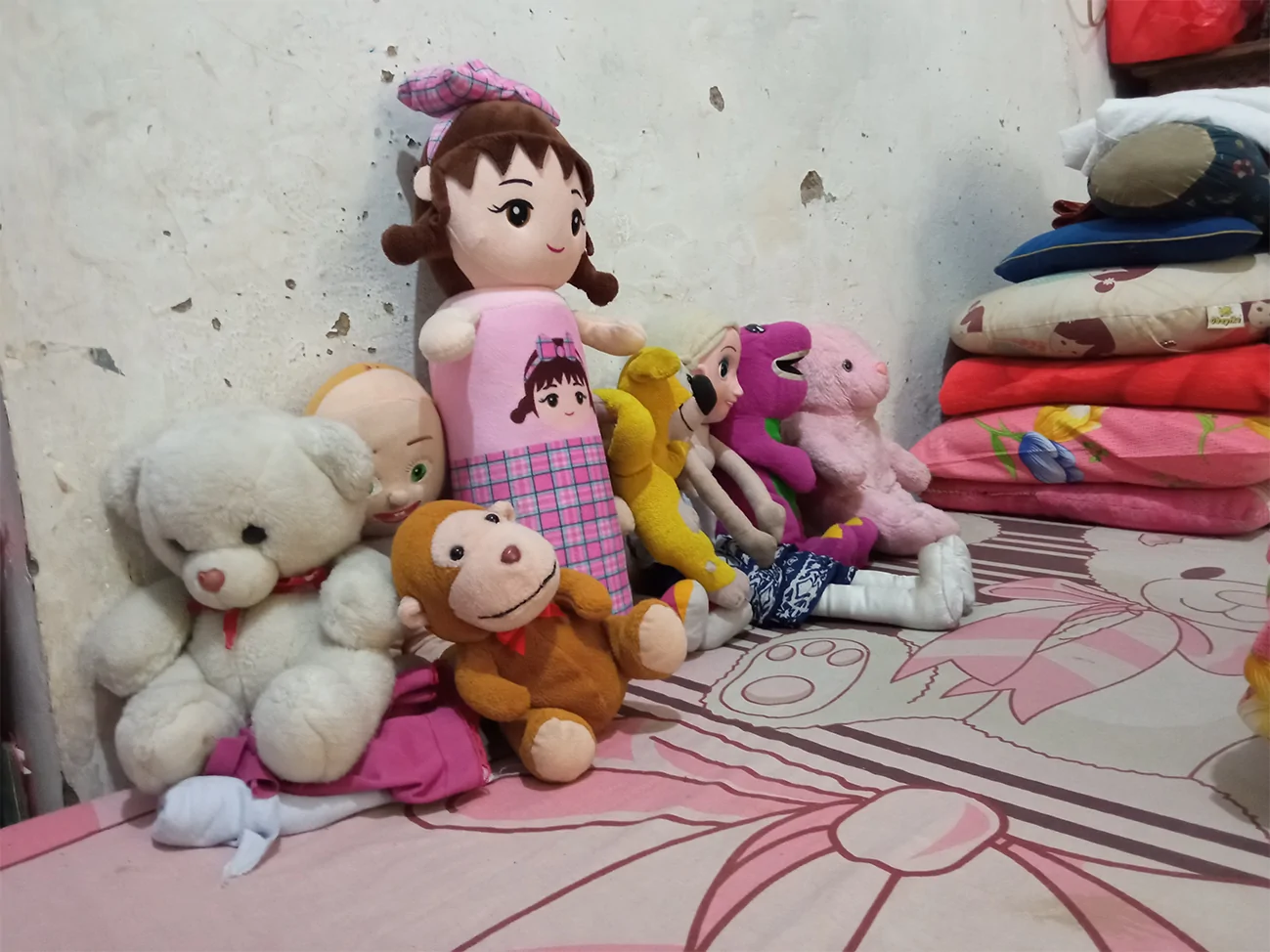
Dolls positioned on the mattress.

The sibling explained that the photo of the dolls lined up in Figure 3 symbolizes the closeness of the sibling relationship with deafblind teenagers, who support each other in the family. Closeness and attachment built from the moment they are born and live together in the family will persist throughout their lives, serving as an adaptive strategy to maintain resilience. On the other hand, they also experience feelings of isolation due to the stigma of a social environment that does not understand the condition of deafblindness.

***Subtheme: Future and anxiety***

The worries of siblings in both towns and villages are characterized by anxiety over their future and the long-term care of their deafblind siblings.

*“I love my sister, but sometimes I am tired and confused too. I don’t know if my marriage will be good, because in the future I will definitely bring it into my little family.” (Participant 1).*

This anxiety causes a heavy burden of anxiety, especially when they have to accompany their younger siblings when their parents work in the fields or away from home. However, behind this burden, siblings in the countryside also exhibit a strong form of resilience, influenced by the cultural values of mutual cooperation and the surrounding environment’s religious beliefs.

### Theme 3: Urban dan Rural divide

The d theme emerges, which includes 3 sub-themes, as siblings reported significant differences in services, facilities, paid work, and sibling support communities in rural and urban areas.

***Subtheme: Health and vocational services***

**Figure 4.**
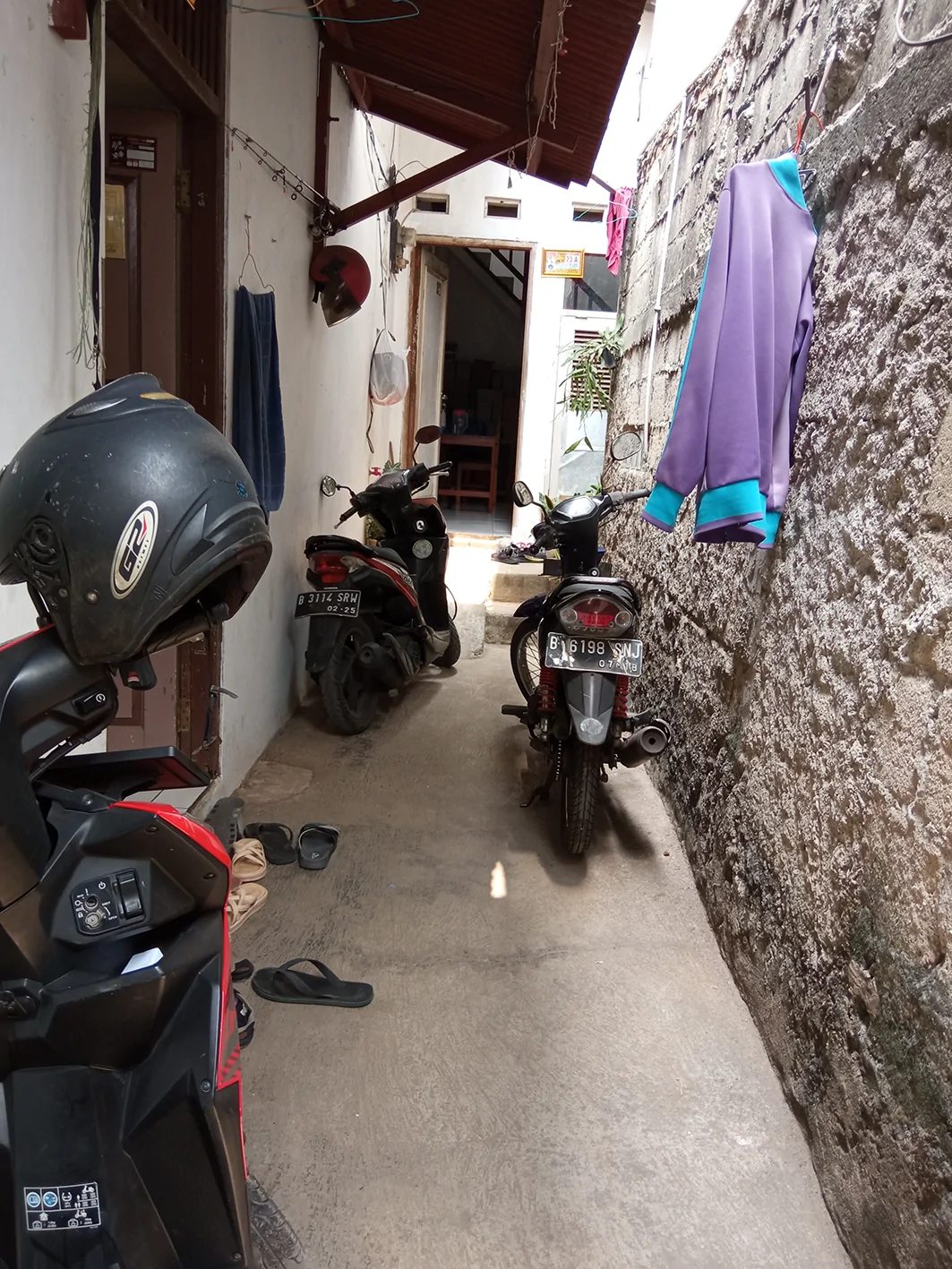
A narrow road to the house entrance.

The city’s siblings reported that health services were adequate but less disability-friendly. Long queues and paperwork bureaucracy can leave teens feeling overwhelmed, deaf, blind, and prone to tantrums and destruction. This situation makes the family prefer vocational services. Figure 4 illustrates access to the alley from densely populated housing areas in the city. The alley can only be passed by 1 motorcycle. This condition makes families have to spend more money when deafblind adolescents need treatment, using a car to access health services.

“… *Once I had to rent a stretcher and 4 people to take my little brother to the big street to access the ambulance, because we lived in a narrow and crowded alley.” (participant 3).*

***Subtheme: Paid work***

Although in the city, living in a densely populated location, this situation is severe when deafblind teenagers often get sick. Siblings often feel that they must support their family’s finances by working part-time to help their parents. The period of transition is acknowledged by siblings to be a time of deteriorating health, which requires additional costs. Siblings living in villages and cities share concerns about the sustainability of their lives in the future. Parents can no longer be relied upon to support the family’s finances, while siblings must consider taking on additional paid work.

*“My parents want me to focus on studying, but I also have to support the family economy. Sometimes I feel like I’ve failed at both.*” *(Participant 6)*.

***Subtheme: Siblings’ support***

**Figure 5.**
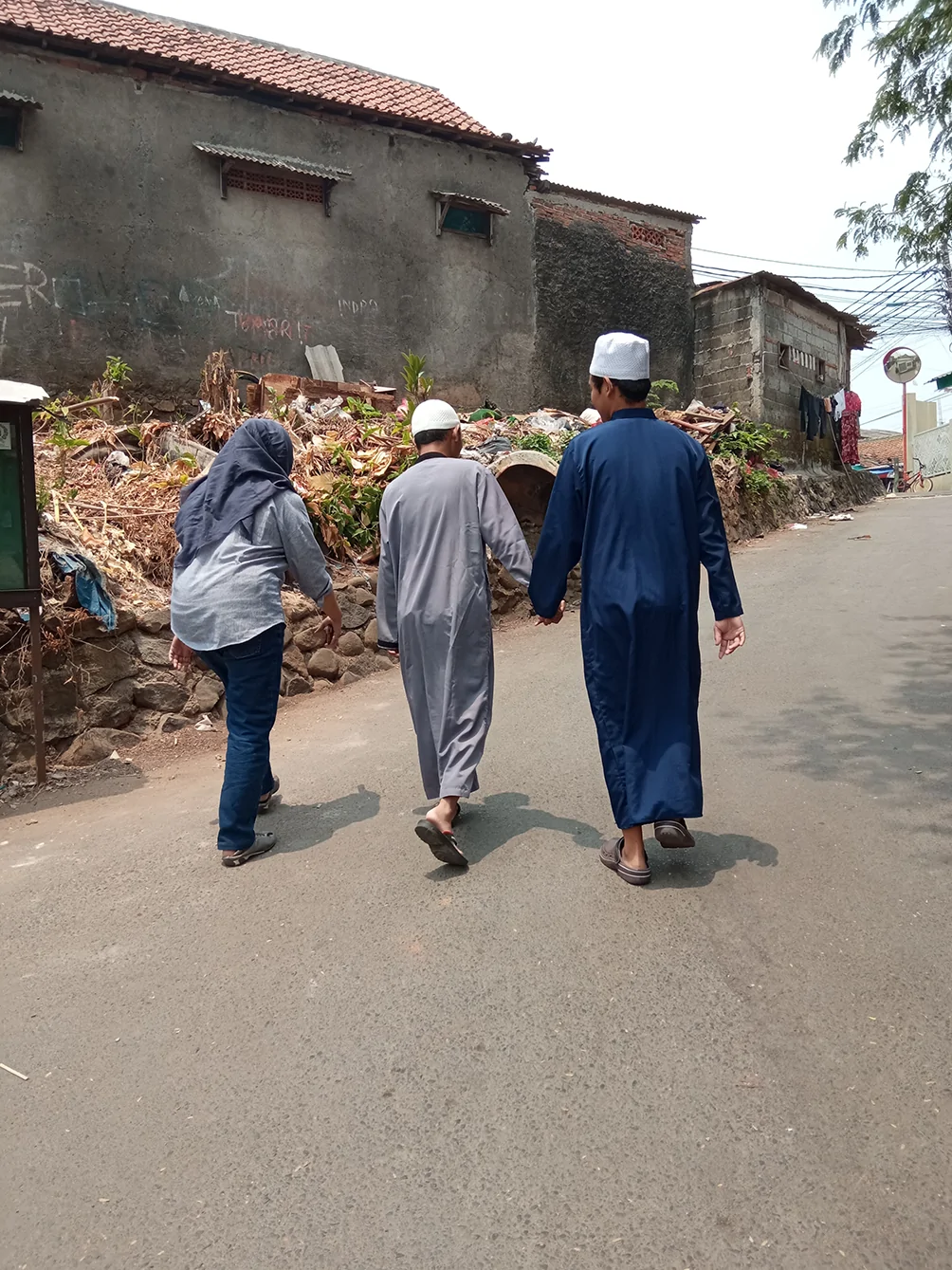
Siblings accompany deafblind teenagers to the mosque for worship.

Sibling community support in urban areas is facilitated by schools serving students with various types of disabilities. Siblings report that this community is very helpful, but siblings feel that they remain alone. Figure 5 shows siblings going to the mosque to pray together. The sibling, who lives in a village, explained that the routine of going to the mosque sometimes causes the deafblind adolescent to throw tantrums due to the crowds. However, the male sibling revealed that there is an inherent obligation as a Muslim to accompany his younger sibling (a deafblind adolescent) as a form of support.

Meanwhile, in rural areas, there is no support community for siblings. This situation makes siblings feel more marginalized and marginalized without support.

*“I don’t find a support community here (village), I don’t find it in cyberspace, I feel increasingly isolated and down” (Participant 4).*

All siblings acknowledged the lack of support for expressing their feelings and anxieties within certain communities in society.

### Theme 4: Social relationships and stigma

The fourth theme highlights how sibling experiences are shaped not only by the family’s internal relationships but also by their interactions with the social environment. It includes 2 sub-themes as follows.

***Subtheme: Sibling identity***

Sibling identities are influenced by the extent to which they feel accepted or rejected due to the stigma associated with their deafblind sibling’s condition. Although various forms of verbal and non-verbal stigma are inherent because they have deafblind siblings, strong support is also found in the environment.

*“Alhamdullilah, there are still people who care, at least the neighbors in front of my house like to ask how my brother is doing,” (Participant 5).*

***Subtheme: Support and solidarity***

Siblings reported feeling socially excluded. They often felt “on the edge and loneliness” in their peers’ social world.

… *“Honestly, I sometimes feel alone, because the environment doesn’t care and my mom always focuses on him (deafblind).” (Participant 3).*

*“When I brought my brother, I was often reprimanded. Because it is troublesome and noisy if I have a tantrum, I feel embarrassed and guilty…” (Participant 6).*

Support more often comes from small circles, such as close friends or teachers who understand their condition. But this support is not always consistent. Meanwhile, rural siblings describe a solidarity that is felt more when the village community knows the family personally.

### Theme 5. Temporal transitions

The fifth theme highlights the shift in time and the role that siblings experience when accompanying deafblind adolescents in the transition phase. It includes 3 sub-themes as follows

***Subtheme: Transition from the school environment to the home***

Siblings in both rural and urban areas report losing their balance. Siblings often feel their routine is “deprived” of having to be involved in treatment immediately.

*“At school, there are teachers and friends who help. But once I got home, I had to stay with him.” (Participant 1).*

***Subtheme: Sibling transition to become the main caregiver***

**Figure 6.**
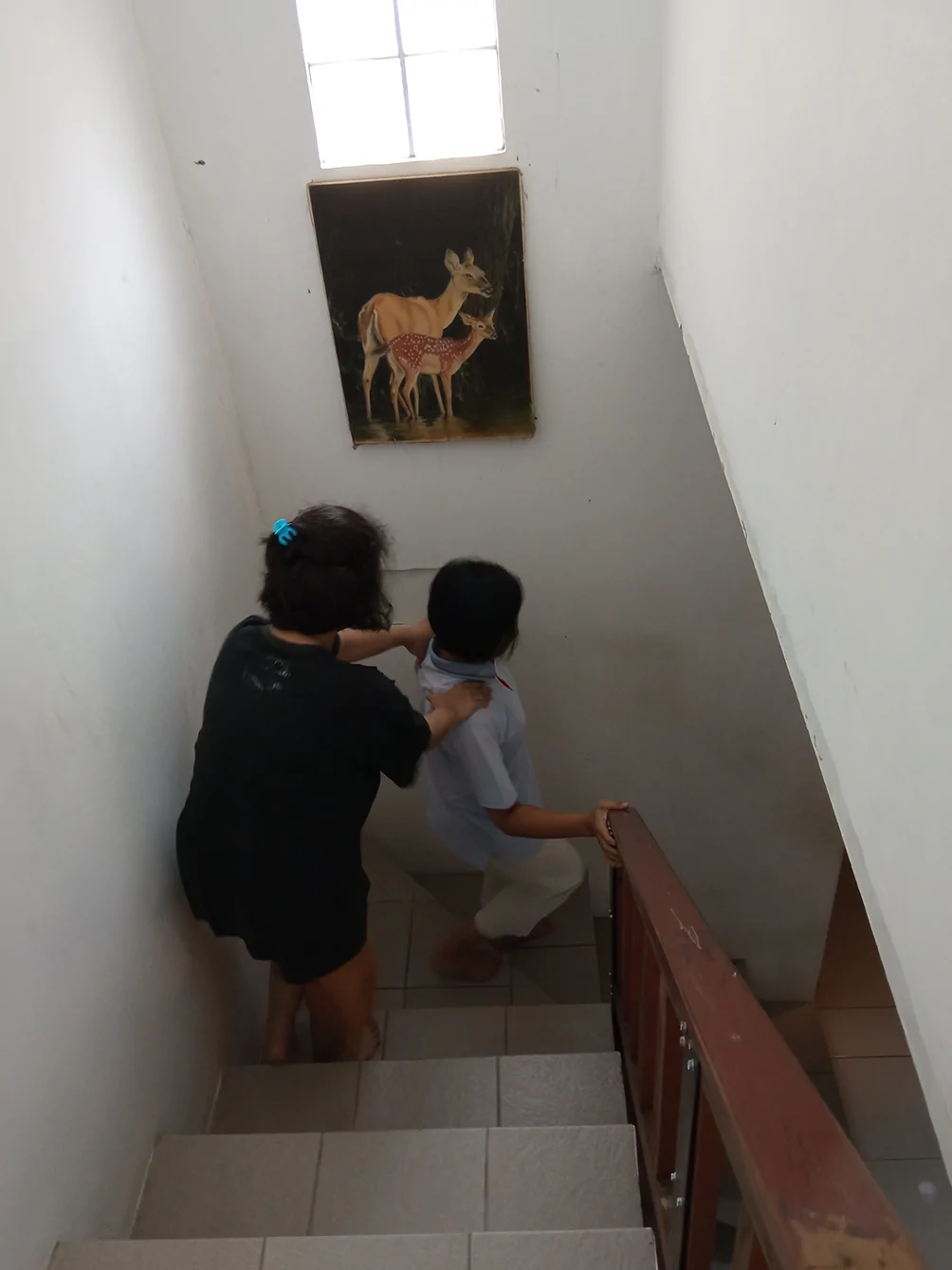
A deafblind teenager always follows her mother and siblings' activities.

Figure 6 illustrates the activities of deafblind teenagers at home, who often prefer to be with their siblings and mother. At school, they are taught to be independent, but at home they always want help and to be accompanied. This role transition is recognized by siblings as a significant burden because they feel that they lack sufficient preparation to assume a new role with complex dynamics throughout the day.

*“I have no choice but to avoid it, so I just go through this process because providing parenting to teenagers like this is not easy,”*

*(Participant 8)*.

**Figure 7.**
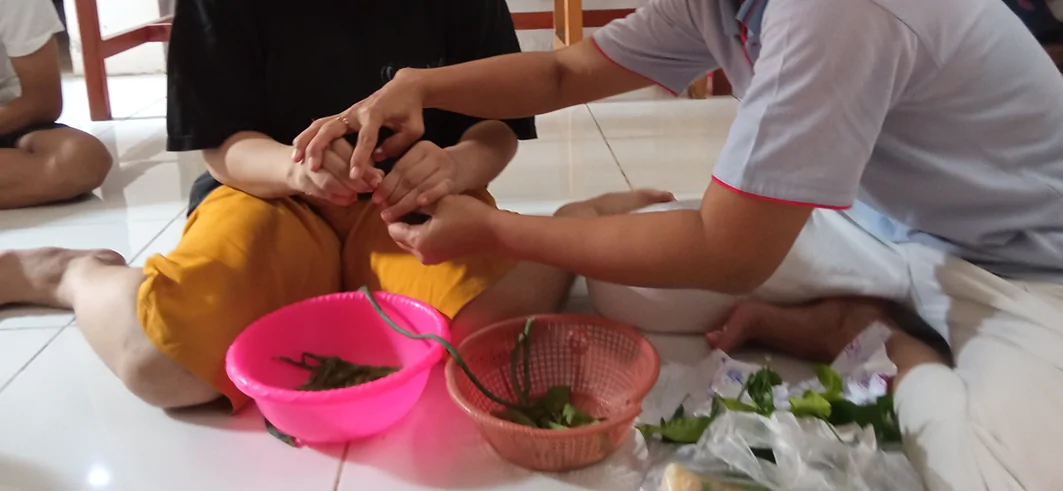
A deafblind teen learns to cut long beans by hand.

Figure 7 shows a deaf-blind teenager chopping vegetables before cooking. This activity takes quite a long time. Siblings stated that all activities involving deaf-blind teenagers require patience and are tailored to their abilities. The process of adapting to home life often leaves siblings impatient and inclined to take on the role of caregiver. Role changes are more accepted as a natural responsibility. Cultural, familial, and religious values lead them to interpret this shift as a moral obligation. However, the physical and psychological burden is still felt.

*“Since I was a child, I have been used to helping. It doesn’t feel weird, but it’s still heavy.” (Participant 7).*

***Subtheme: Uncertain future***

Siblings talk about career and educational anxiety, and their relationship with a potential partner later in life. They are worried that they will have to sacrifice future opportunities because they have to take care of their younger siblings with special needs.

*“If I go to college or work out of town, who will take care of my sister?” (Participant 3).*

### Theme 6. Bridging advocacy

The sixth theme, Bridging Advocacy, highlights the role of siblings as vital bridges connecting deafblind youth with their families, institutions, and communities. It includes two sub-themes.

***Subtheme: Communication liaison***

Siblings in rural areas acknowledged that this was a good way to communicate the presence of deafblind teenagers and their uniqueness within their homes. Meanwhile, city siblings reported that they often play a role in communicating the fulfillment of service needs to health providers and the community in the area. Siblings are the link between language, feelings, and needs bringing voice to spaces that are often closed to people with multiple disabilities. Urban siblings serve as information liaisons to ensure that services can be provided.

*“If I go to the doctor, I will talk to you, because parents sometimes lack confidence and stutter.*” *(Participant 4)*.

**Subtheme: Hidden liability**

In rural areas, the role of siblings focuses more on social responsibility and community interaction to turn stigma into solidarity, so that their function resembles that of “local social agents.”

*“Even though I wasn’t invited, I always invited my deafblind brother to join the Independence Day celebrations in the village. People often looked at my brother strangely, but that was my chance to explain to them about my brother.” (Participant 6).*

## Discussion

In the study of disability, the intersection of sibling experiences in adolescent deafblindness in the transition phase is an important area and is rarely disclosed. Sibling experiences are often difficult to fully capture using traditional methods. The use of the photovoice method in this study played an important role in uncovering the dynamics of marginalized groups, such as siblings who grow up together with adolescent deafblindness, accompanied by mental and intellectual barriers. This reinforces the findings that affirm this approach presents embodied knowledge, reflecting sibling experiences in a deep, participatory, and visually meaningful way. ^46, 51^

Through this methodology, this study uncovered findings about the experiences and roles of adolescent siblings with deafblindness, accompanied by mental and intellectual barriers, with complex and previously uninvolved outcomes. These findings confirm the study, ^40^ which found that the presence of individuals with deafblindness impacts families, including siblings, although this is rarely acknowledged. According to, ^27^ the positive and negative impacts that arise from siblings stem from their being the ones with the longest relationship. Siblings are family members who live together in the long term and have the longest-lasting emotional bond. ^52, 53^

Our findings indicate that, during the transition phase, both adolescents with deafblindness and their siblings experience a critical period. In line with ^15^ emphasized that transition is not only a matter of individuals with disabilities but also of all family members involved. While previous studies still highlight the important role of parents and lack the role of siblings. ^12, 15, 23, 6, 16^

This crucial situation has been discussed in other studies that confirm that the end of structured support from school is often not followed by the availability of follow-up services, so that the family becomes the main focus and bears the dual role. ^12, 21, 54, 55^ The study ^56^ confirms these findings by highlighting the weakening of collaboration and support after deafblind high school graduation, leading siblings to assume a dual role.

The fact that there is no collaboration among stakeholders to support deafblindness adolescents often causes families to have difficulty accessing the support needed by them after graduating from high school. ^4, 56^ This is in line with ^13^ While previous studies highlighted the limitations of post-school services, this study offers a new perspective: siblings are often the primary caregivers when professional caregivers are absent, especially in rural areas with limited access to services. As a result, the welfare of all members has declined due to a lack of support and weak policies for this group.

In Indonesia, sibling parenting is deeply rooted, especially in multigenerational extended families. The findings of invisible care work in this study reveal that, despite being central actors who routinely assume a parenting role, siblings are often seen as a family’s moral obligation rather than a valuable domestic role. Although rural siblings acknowledge that the village’s kinship system remains strong, long-term parenting remains an inevitable burden. Deafblind adolescents will, in fact, continue to need the help of others throughout their lives because of the complexity they experience. Siblings take on a variety of roles and responsibilities to support siblings with severe disabilities. ^57^ When one sibling has a disability, the relationship can become more complex, and siblings play multiple roles. ^53, 58^ Similar to our findings, ^53^ found that even in developed countries, siblings also serve as caregivers, in addition to other roles they must fulfill. In fact, there are only a few protocols that guide providing guidance in sibling care and assistance to siblings with disabilities. ^59^

Emotional strain is a finding often discussed among siblings in this study. Siblings have the longest emotional and familial bonds ^52^ but often fluctuate over time, making relationships tense and difficult. ^59^ All participants in this study emphasized that they felt a sense of exclusion and insecurity because they were not prepared to face obstacles in establishing relationships outside their community. Similar to these findings, ^40^ describe that siblings living with deafblindness face psychological and social challenges, including emotions such as feelings of neglect, resentment, shame, jealousy, and anxiety.

Participants in this study also revealed that geographical location, culture, gender, and values are influential factors in determining social relationships and stigma. This study builds on the findings of ^16^ regarding the transition gap resulting from differences in service access. Geographical and cultural contexts strongly determine sibling experiences. ^11^ These findings also reinforce that, although social stigma is a significant barrier to sibling inclusion, rural social contexts can provide opportunities for kinship and community solidarity.

The study also showed that how siblings manage emotions is influenced by cultural context. In cities, siblings experience identity conflicts, whereas in the countryside, they often interpret emotional burdens through spirituality and collective values. This perspective expands on the findings ^60^ on the importance of family communication in building resilience. ^39, 61, 62^ reveal emotional ambivalence in families with multiple disabilities, facing more real identity crises in the transition phase. The concept of resilience provides insight into the different responses of each sibling. ^27^

Bringing advocacy into this study is an interesting finding. They demonstrate a crucial, multi-pronged advocacy role in supporting their siblings, though a small number admit to feeling powerless. This reinforces that siblings perceive their advocacy role in very different ways in each region (e.g., sibling supporters, contact persons, advocates in the event of bullying, service information connectors, and reducing negative voices from the community about disabilities. ^53^ Regardless of the type of advocacy, all siblings felt they needed more information and real peer support, ^63^ and future planning. ^64^

These findings underscore the need for interventions to enhance the well-being of siblings of adolescents with deafblindness during the transition period. The impact of mental health and improved well-being needs to be considered for a comprehensive, needs-based intervention to meet their needs. ^65, 66^ The study also underscores the frequent overlook of siblings in transition planning programs conducted by schools and parents. ^67^ It also suggests that a significant gap exists in how siblings are often overlooked in terms of recognition.

### Study limitation

This study makes a significant contribution to strengthening the role and voice of deafblind adolescents in Indonesia, but several limitations should be acknowledged. First, the issues studied focus on the transition from school to family. While this focus is important, it also limits the scope for further exploration. The experiences of siblings from childhood through early adulthood are not thoroughly examined, so a comprehensive understanding of their roles and trajectories remains limited. Additionally, gender, socioeconomic status, and cultural expectations are mentioned but not deeply explored to understand how these factors interact to shape sibling roles.

## Conclusion and practical implications

The findings show that siblings often play an invisible but crucial role, ranging from providing emotional support and day-to-day parenting to advocacy and serving as social mediators for their deafblind siblings during the transition. This study shows how cultural, economic, and geographical factors (urban vs. rural) affect sibling engagement. Although the results cannot be generalized, this study confirms the need for multidimensional interventions. In terms of practical implications, first, the transition planning policy in special needs education must include siblings’ involvement to ensure adequate family support after adolescents graduate from school. Second, social workers in schools play a crucial role in connecting students’ interests with the broader ecosystem outside of school, including family members and various service systems that can be optimized to support the independence of adolescents with deafblindness. Third, family support must be strengthened by enhancing the basic capacity of parenting and mentoring, as well as peer support that enables siblings to alleviate psychological pressure and feelings of isolation. Finally, this study confirms the importance of follow-up studies with longitudinal designs and more diverse cross-regional and cross-cultural samples in developed and developing countries.

## Data Availability

Complementary data supporting the findings of this study are available from the authors of the correspondence upon reasonable request. The data from these findings is limited due to the institution’s confidentiality policy regarding information, data, and photos from participants, parents, and the school. The main qualitative data collected and analyzed in this study were not publicly accessible due to the sensitive, confidential nature of the photographic material, which could potentially identify participants. Researchers seeking access to anonymized data must submit a written request to the first author at hastin@binawan.ac.id. Access will be granted only upon approval by the Research Ethics Committee, Department of Social Welfare Sciences, Faculty of Social and Political Sciences, University of Indonesia (No. S-845/UN2. F9.D1/PPM.00.04/2023).
