# Unseen Care, Unequal Worlds: Photovoice Narratives of Siblings Supporting Deafblind Adolescents in Indonesia

**DOI:** 10.12688/f1000research.171949.2

**Published:** 2026-07-13

**Authors:** Hastin Trustisari, Fentiny Nugroho, Dini Widinarsih, Muhammad Arsyad Subu, Richard Mottershead

**Affiliations:** 1 Department of Social Welfare, Faculty of Social and Political Sciences, University of Indonesia, Depok, Indonesia; 2 Department of Social Welfare, Faculty of Business and Social Science, Universitas Binawan, Jakarta, Indonesia; 3 Department of Nursing, College of Health Sciences, University of Sharjah, Sharjah, United Arab Emirates; 4 Faculty of Nursing and Midwifery, Universitas Binawan, Jakarta, Indonesia; 5 SEHA, Sakina, Behavioural Science Institute, Al Ain, United Arab Emirates; 6 College of Nursing, University of Baghdad, Baghdad, Iraq

**Keywords:** Deafblindness; siblings; photovoice; transition; social work; Indonesia.

## Abstract

**Background:**

Congenital deafblindness is a rare and complex condition characterised by combined hearing and vision impairment, frequently accompanied by additional intellectual, developmental, and psychosocial disabilities. Adolescents with deafblindness often encounter substantial challenges during the transition from school to adulthood, particularly in low-resource settings where formal disability services are limited. While existing research has predominantly focused on parental experiences, the perspectives of siblings—who frequently assume significant caregiving and advocacy responsibilities—remain underrepresented, especially within family-centred contexts such as Indonesia.

**Aim:**

To explore the lived experiences and caregiving roles of siblings supporting adolescents with congenital deafblindness and additional intellectual and mental disabilities during post-school transition in rural and urban Indonesia.

**Methods:**

A qualitative participatory Photovoice design was employed. Eight siblings aged 15–25 years were purposively recruited through special education schools in rural and urban Indonesia. Over a 12-week period, participants documented their caregiving experiences through photography, semi-structured interviews, and focus group discussions. Data were analysed using reflexive thematic analysis.

**Results:**

Four interconnected themes were identified: (1) invisible caregiving and role transformation; (2) emotional burden, resilience, and uncertain futures; (3) geography, stigma, and unequal support systems; and (4) siblings as communication brokers and hidden advocates. Across both rural and urban settings, siblings undertook extensive caregiving, advocacy, and emotional support responsibilities that were largely unrecognised within formal transition planning. Urban participants described tensions between education, employment, and caregiving demands, whereas rural participants reported stronger kinship support alongside limited access to specialist services and persistent disability stigma.

**Conclusions:**

Siblings play a critical yet frequently overlooked role in supporting adolescents with congenital deafblindness during post-school transition. Caregiving experiences were shaped by the interaction of geographical context, cultural expectations, family structures, and service availability. The findings position siblings as critical yet frequently overlooked contributors to transition support and extend current understanding of deafblindness by foregrounding sibling perspectives within a low-resource, family-centred context. Greater recognition of sibling caregiving within disability policy, transition planning, and family-centred interventions is required. Future longitudinal and cross-cultural research is required to better understand how sibling roles evolve across the life-course and across diverse care contexts.

## Introduction

### Understanding Deafblindness and Additional Disabilities

Deafblindness is a distinct and highly heterogeneous disability characterised by a combined impairment of vision and hearing that significantly affects communication, access to information, mobility, social participation, and independent living ( Dammeyer, 2012; World Federation of the Deafblind [WFDB], 2018). Unlike single sensory impairments, deafblindness creates unique barriers that cannot be fully understood as the simple addition of hearing and vision loss. Rather, the interaction of these impairments often results in complex communication needs and increased reliance on environmental, familial, and community support systems ( Bruce et al., 2018; Nelson & Bruce, 2016).

Deafblindness may be congenital or acquired. Congenital deafblindness is present at birth or develops during early childhood and is frequently associated with additional intellectual, developmental, neurological, or psychosocial disabilities. Conditions such as CHARGE syndrome, congenital infections, genetic disorders, cerebral palsy, autism spectrum disorder, and intellectual disability are commonly reported among individuals with congenital deafblindness ( Bruce et al., 2018; Dammeyer, 2012). In contrast, acquired deafblindness often develops later in life through ageing, injury, illness, or progressive sensory conditions and may not necessarily involve additional disabilities. Recognising this distinction is important because support needs, communication approaches, educational experiences, and family caregiving demands differ substantially across deafblind populations.

Globally, approximately 0.2% of the population experiences severe forms of deafblindness, while milder forms affect an estimated 2% of individuals worldwide ( WFDB, 2018). Although relatively uncommon, deafblindness has been recognised as a population at risk of social exclusion due to barriers in communication, service access, education, employment, and civic participation ( Simcock, 2014, 2017). The relative invisibility of deafblindness within public discourse and policy development has contributed to the marginalisation of this population within health, education, and social care systems, despite the substantial support needs often associated with the condition ( Roy et al. 2019; Simcock, 2014).

The implications of deafblindness extend beyond the individual to the wider family. Families frequently assume long-term caregiving responsibilities that encompass communication support, personal care, advocacy, supervision, educational engagement, and navigation of complex service systems ( Correa-Torres & Bowen, 2016). These responsibilities may generate emotional, practical, financial, and relational challenges, particularly when formal support services are limited. While parental experiences have received increasing scholarly attention, siblings remain one of the least visible groups within deafblindness research. Yet siblings frequently provide sustained emotional, practical, communicative, and advocacy support across the life course and may ultimately become primary caregivers as parents age. Understanding sibling experiences is therefore essential not only for appreciating family adaptation to deafblindness but also for informing future models of family-centred transition planning, disability support, and long-term care.

### Post-School Transition and Family Caregiving

Transition to adulthood represents a critical developmental period for young people with disabilities. Within deafblindness research, transition commonly refers to the movement from school-based educational environments into adult life, including employment, community participation, independent living, health care systems, and long-term support arrangements ( Lipscomb et al., 2017; Zatta & McGinnity, 2016). Successful transition requires coordinated planning across educational, health, social care, vocational, and family systems. However, for many adolescents with deafblindness, this transition is characterised by discontinuity of services, reduced social support, uncertainty regarding future opportunities, and increased dependence on family caregivers ( McDonnall & Cmar, 2018; Petroff et al., 2019).

Research consistently demonstrates that young people with deafblindness experience poorer post-school outcomes than many other disability groups, including lower rates of employment, independent living, and community participation ( Cmar et al., 2018; Petroff et al., 2019). The conclusion of formal educational support often creates a service gap in which responsibility for ongoing care and advocacy shifts increasingly towards families. Consequently, transition is not solely experienced by the individual with deafblindness but by the entire family system, including parents and siblings who frequently assume new responsibilities and roles ( Francis et al., 2018; Trainor et al., 2020).

Existing studies have primarily focused on parental concerns regarding transition planning, service coordination, and future care arrangements ( Correa-Torres & Bowen, 2016; Zatta & McGinnity, 2016). While these perspectives are important, they provide only a partial understanding of family transition experiences. Siblings frequently maintain lifelong relationships with individuals with disabilities and often become future caregivers, advocates, decision-makers, and sources of emotional continuity. Consequently, understanding sibling experiences is central to understanding how families adapt to disability-related transitions across the life course. Despite this, sibling perspectives remain comparatively absent from deafblindness transition research.

### Sibling Caregiving, Invisible Labour, and Family Relationships

Sibling relationships are among the longest-lasting relationships experienced across the life course. For siblings of individuals with disabilities, these relationships frequently extend beyond traditional familial roles to include caregiving, advocacy, supervision, emotional support, and future planning responsibilities ( Hall & Rossetti, 2018; Lee & Burke, 2018). Research across disability contexts suggests that siblings often undertake significant responsibilities that remain largely unrecognised by formal service systems and policy frameworks ( Bigby et al., 2015; Strohm, 2018).

The concept of invisible care work provides a useful lens through which to understand sibling experiences. Invisible care work refers to unpaid, often unacknowledged caregiving activities that occur within everyday family life, including personal care assistance, emotional labour, communication support, household management, supervision, and advocacy. Such responsibilities may influence educational opportunities, employment aspirations, social participation, identity development, and psychological wellbeing ( Arcous et al., 2024; Giallo et al., 2012). While some siblings describe caregiving as a source of meaning, resilience, and personal growth, others report experiences of burden, uncertainty, social isolation, and concern regarding future responsibilities ( Burke et al., 2015; Heller & Arnold, 2010).

Recent evidence suggests that siblings of individuals with deafblindness may experience distinctive challenges associated with communication barriers, lifelong support needs, and limited societal understanding of the condition ( Arcous et al., 2024). Nevertheless, the perspectives of siblings remain substantially underrepresented within deafblindness research. This omission is particularly significant during the post-school transition period, when caregiving demands, family expectations, and future planning responsibilities often intensify. From a family perspective, sibling caregiving can be understood as part of a dynamic process of family adaptation in which responsibilities, identities, and relationships are continually renegotiated in response to changing care needs. Examining sibling experiences therefore provides insights not only into caregiving practices but also into broader processes of resilience, role transition, and family functioning across the life course.

### The Indonesian Context: Family Caregiving, Geography, and Disability

The experiences of siblings supporting adolescents with deafblindness are shaped not only by disability-related factors but also by broader cultural, social, and geographical contexts. In Indonesia, family caregiving remains the dominant model of support for individuals with disabilities. Although social attitudes and service provision have gradually evolved, care responsibilities continue to be strongly embedded within family systems, particularly within multigenerational households where caregiving is often shared among parents, siblings, grandparents, and extended relatives ( Yulaswati et al., 2021).

Within Indonesian society, values associated with collective responsibility, filial obligation, reciprocity, and family solidarity frequently influence caregiving expectations. These cultural norms can provide important sources of support and resilience for families caring for individuals with disabilities. At the same time, they may contribute to the normalisation of caregiving responsibilities among siblings, rendering their contributions less visible and less likely to be recognised by formal support systems. In many households, siblings are expected to assist with supervision, communication, transportation, household responsibilities, and personal care as part of everyday family life.

Geographical inequalities further influence the experiences of families affected by disability. Significant differences continue to exist between urban and rural regions in terms of access to healthcare, specialist educational services, vocational opportunities, disability support programmes, transportation infrastructure, and community resources ( Yulaswati et al., 2021). Urban environments may offer greater access to specialist services and educational opportunities but are often characterised by social fragmentation, economic pressures, and reduced community support networks. Conversely, rural communities may provide stronger kinship connections and social cohesion while simultaneously experiencing shortages of specialist disability services and professional support.

Disability stigma also remains a significant challenge within many communities. Families may encounter misconceptions regarding disability, social exclusion, discrimination, or reduced opportunities for participation in community life. Such experiences can influence not only individuals with disabilities but also their siblings, who may experience secondary stigma, social isolation, or altered identity formation because of their association with a family member with a disability ( Arcous et al., 2024). Understanding how these cultural and geographical factors intersect with caregiving responsibilities is therefore essential for developing a more nuanced understanding of sibling experiences within Indonesia. Consequently, Indonesia provides an important context for exploring how culture, geography, disability, and family caregiving intersect to shape transition experiences.

### Photovoice and Participatory Disability Research

Photovoice is a participatory visual research methodology that enables participants to document, reflect upon, and communicate their lived experiences through photography and dialogue ( Wang & Burris, 1997). Grounded in participatory action research principles, Photovoice seeks to democratise knowledge production by positioning participants as active contributors rather than passive subjects of research. Through the creation and discussion of photographs, participants are able to identify issues of importance, share personal narratives, and generate insights that may be difficult to access through conventional qualitative methods alone.

Photovoice has been increasingly used within disability research to amplify marginalised voices, facilitate self-expression, and explore complex social experiences that are often overlooked within traditional research approaches ( Chinn & Balota, 2023; Cluley, 2016). The visual and participatory nature of the method is particularly valuable when investigating hidden forms of caregiving, emotional labour, family dynamics, and everyday experiences that may be difficult to articulate verbally. By encouraging participants to document their daily realities through images, Photovoice allows researchers to access embodied, contextual, and emotionally nuanced dimensions of experience.

The use of Photovoice in the present study was particularly appropriate because sibling caregiving often occurs within private family spaces and may remain largely invisible to professionals, policymakers, and wider society. Through photographs, interviews, and group discussions, participants were able to represent aspects of their caregiving roles that might otherwise remain hidden. Furthermore, the collaborative nature of Photovoice aligned with the study’s commitment to recognising siblings as experts in their own experiences and as important contributors to disability knowledge.

Although Photovoice has been used to explore the experiences of individuals with intellectual disabilities, autism, and other disability groups, its application to sibling experiences within deafblindness remains limited ( Chinn & Balota, 2023; Pavlopoulou & Dimitriou, 2020). The methodology therefore aligned closely with the study’s aim of foregrounding sibling voices and generating participant-led understandings of transition and care.

### Research Gap

Existing research demonstrates that families play a central role in supporting adolescents and young adults with deafblindness, particularly during the transition from school to adulthood. However, the majority of studies have focused on parental experiences, service provision, educational outcomes, or the perspectives of individuals with deafblindness themselves ( Correa-Torres & Bowen, 2016; Zatta & McGinnity, 2016). Comparatively little attention has been given to siblings, despite evidence suggesting that they frequently assume significant caregiving, advocacy, and emotional support responsibilities.

Recent reviews have highlighted the psychological, social, and relational impacts experienced by siblings of individuals with deafblindness, while simultaneously identifying substantial gaps in the evidence base ( Arcous et al., 2024). Research exploring sibling experiences during post-school transition remains particularly limited. Furthermore, most existing studies originate from high-income countries with relatively well-developed disability support systems, limiting understanding of sibling experiences within low-resource and family-centred contexts.

To date, little is known about how siblings navigate caregiving responsibilities during post-school transition within Indonesia, where family-based support remains the primary source of care and where significant geographical disparities influence access to services and opportunities. Equally absent are studies that compare sibling experiences across rural and urban settings while employing participatory methodologies capable of capturing the complexity of everyday caregiving realities. Addressing this gap is important not only for informing disability support and transition planning in Indonesia but also for advancing international understanding of sibling caregiving within low-resource and family-centred care systems.

### Aim and Research Question

This study aimed to explore how siblings experience, interpret, and negotiate caregiving responsibilities during the post-school transition of adolescents with congenital deafblindness and additional intellectual and mental disabilities in rural and urban Indonesia. Particular attention was given to how caregiving roles are shaped by family relationships, geographical context, cultural expectations, and access to formal support systems.

By foregrounding sibling perspectives, the study sought to address a significant gap in the deafblindness literature and contribute to a more nuanced understanding of family-centred transition experiences within low-resource settings.

The study was guided by the following research question:

− How do siblings experience and negotiate caregiving responsibilities during the post-school transition of adolescents with congenital deafblindness and additional intellectual and mental disabilities in rural and urban Indonesia?

## Methodology

### Research Design

This study employed a qualitative participatory research design using Photovoice methodology. Photovoice is a participatory visual approach that enables individuals to document and communicate aspects of their lived experiences through photography, dialogue, and critical reflection ( Wang & Burris, 1997). Rooted in participatory action research principles, Photovoice seeks to democratise knowledge production by positioning participants as active contributors to the research process rather than passive subjects. Through the creation and interpretation of photographs, participants are able to identify issues of personal significance, reflect upon their experiences, and generate insights that may otherwise remain hidden within everyday life.

The study was informed by an interpretivist epistemological perspective, which assumes that reality is socially constructed and that individuals develop meaning through their interactions with others and their environments. This perspective was particularly appropriate because the research sought to understand how siblings interpreted and negotiated caregiving responsibilities during the post-school transition of adolescents with congenital deafblindness and additional intellectual and mental disabilities. The study also drew upon participatory principles by recognising siblings as experts in their own experiences and actively involving them in data generation, reflection, and thematic development.

Photovoice was selected because sibling caregiving frequently occurs within private family environments and remains largely invisible to service providers, policymakers, and researchers. The methodology provided opportunities for participants to visually document caregiving experiences, emotional challenges, advocacy activities, and everyday realities that may be difficult to express through interviews alone. Previous research has demonstrated the value of Photovoice in disability research for accessing nuanced, contextualised, and embodied experiences while promoting participant voice, agency, and engagement ( Chinn & Balota, 2023; Cluley, 2016; Pavlopoulou & Dimitriou, 2020). Photovoice has been particularly valuable in studies involving marginalised populations because it enables participants to communicate experiences that may be difficult to articulate through verbal accounts alone and facilitates the exploration of social, relational, and environmental dimensions of everyday life ( Sutton-Brown, 2014; Wang & Burris, 1997). Consequently, the methodology aligned closely with the study’s aim of foregrounding sibling perspectives and generating participant-led understandings of caregiving, transition, and family life.

### Researcher Positionality and Reflexivity

The multidisciplinary research team comprised scholars and practitioners with expertise in social work, disability studies, nursing, mental health, and qualitative research. Several members of the research team had extensive experience working with individuals with disabilities, families, and community-based support services. Consistent with contemporary qualitative research guidance, reflexivity was understood as an ongoing process through which researchers critically examined how their professional backgrounds, assumptions, values, and relationships with the research topic could influence data generation, interpretation, and representation ( Berger, 2015; Finlay, 2002).

Reflective discussions were conducted during data collection and analysis to examine assumptions, interpretations, and potential biases. Field notes, analytic memos, and collaborative discussions among researchers were used to support reflexive engagement with the data. Particular attention was given to avoiding deficit-oriented interpretations of disability and caregiving by privileging participant narratives and ensuring that findings reflected both challenges and sources of resilience. Reflexive engagement was therefore used not to eliminate researcher influence but to enhance transparency, credibility, and interpretive rigour throughout the analytic process ( Braun & Clarke, 2021).

### Setting and Context

The study was conducted in rural and urban regions of Indonesia. The selection of both settings was intended to capture the influence of geographical context on sibling caregiving experiences during post-school transition. In Indonesia, disability support remains largely family-centred, although access to educational, health, and social services varies considerably across geographical regions.

For the purposes of this study, urban settings were characterised by greater access to specialist educational and healthcare services, higher population density, and more developed infrastructure. Rural settings were characterised by lower population density, stronger kinship-based community structures, and more limited access to specialist disability services. Including participants from both contexts enabled exploration of how geography shaped caregiving experiences, support systems, advocacy roles, and transition pathways. This comparative approach facilitated a deeper understanding of how social, cultural, and structural contexts influence family responses to disability and transition. Characteristics of adolescents with congenital deafblindness participating in the study are presented in Tables 1 and 2.

**Table 1.** Characteristics of adolescents with congenital deafblindness — urban participants.

Initial name	Age (y.o)	Gender	Degree of hearing loss	Degree of vision loss	Additional disabilities	Communication mode	Educational placement
D1	18	Female	Profound	Profound	Intellectual disability, epilepsy	Sign language, tactile	Non-government school
D2	19	Female	Moderate	Profound	Intellectual disability, autism	Sign language, tactile	Non-government school
D3	20	Female	Severe	Severe	Intellectual dan mental disability	Sign language, tactile	Non-government school
D4	19	Male	Severe	Moderate	Mental disability, cerebral palsy	Sign language, tactile	Non-government school

**Table 2.** Characteristics of adolescents with congenital deafblindness — rural participants.

Initial name	Age (y.o)	Gender	Degree of hearing loss	Degree of vision loss	Additional disabilities	Communication mode	Educational placement
A1	17	Male	Profound	Profound	Intellectual disability	Sign language, tactile	Government school
A2	20	Male	Severe	Profound	Cerebral palsy, intellectual disability	Sign language, tactile	Government school
A3	19	Female	Severe	Severe	Intellectual disability	Sign language, tactile	Government school
A4	19	Male	Moderate	Moderate	Mental dan intellectual disability	Sign language, tactile	Government school

### Participants and Recruitment

A purposive sampling strategy was employed to recruit siblings of adolescents with congenital deafblindness and additional intellectual and mental disabilities. Participants were recruited through special education schools serving students with multiple disabilities. School staff, teachers, and social workers acted as gatekeepers by identifying families who met the inclusion criteria and providing information about the study.

To be eligible, participants were required to:

1. Be aged between 15 and 25 years;
2. Be a sibling of an adolescent with congenital deafblindness and additional intellectual and/or mental disabilities;
3. Reside within the same household as their sibling;
4. Be actively involved in providing support or assistance during the transition from school to adulthood; and
5. Be willing to participate in all phases of the Photovoice process.

Eight siblings participated in the study, comprising four participants from urban regions and four participants from rural regions. Demographic characteristics of participating siblings are presented in Tables 3 and 4.

**Table 3.** Sibling demographic background in urban areas.

Participant	Age	Gender	Position in the family	Family context	Parents’ marital status	Socioeconomic status
1	20	Female	Older sister	Nuclear family	Married	Higher
2	19	Male	Younger brother	Nuclear family	Divorce	Lower
3	25	Female	Older brother	Extended family	Single parent	Higher
4	21	Female	Older sister	Nuclear family	Married	Middle

**Table 4.** Sibling demographic background in rural areas.

Participant	Age	Gender	Position in the family	Family context	Parents’ marital status	Socioeconomic status
5	16	Female	Younger sister	Nuclear family	Married	Higher
6	17	Male	Younger brother	Nuclear family	Married	Lower
7	25	Female	Older sister	Extended family	Divorce	Higher
8	23	Female	Older sister	Extended family	Single parent	Middle

The sample included both male and female participants and represented diverse family structures and socioeconomic backgrounds.

Rather than seeking statistical representation, the study prioritised depth, richness, and contextual understanding of sibling caregiving experiences. Purposive sampling was considered appropriate because participants possessed direct experience of the phenomenon under investigation and were therefore uniquely positioned to provide detailed insights into caregiving during post-school transition. The sample size was deemed suitable for an intensive Photovoice study because participants generated multiple forms of data, including photographs, interviews, focus group discussions, field reflections, and collaborative thematic discussions. Consistent with the concept of information power, sample adequacy was determined by the specificity of the study aim, the depth of participant experiences, the quality of dialogue generated, and the richness of the resulting dataset rather than numerical considerations alone ( Malterud et al., 2016). Similar sample sizes have been reported in participatory and Photovoice studies exploring complex lived experiences within marginalised populations ( Sutton-Brown, 2014; Wang & Burris, 1997).

### Ethical Considerations

Ethical approval was obtained from the Research Ethics Committee of the Department of Social Welfare Sciences, Faculty of Social and Political Sciences, University of Indonesia (No. S-845/UN2.F9.D1/PPM.00.04/2023).

Written informed consent was obtained from all participants aged 18 years and above. For participants younger than 18 years, written parental consent and participant assent were obtained prior to participation. Families were informed of the study objectives, procedures, potential risks, confidentiality arrangements, and their right to withdraw at any time without consequence.

Given the visual nature of Photovoice, particular attention was paid to photographic ethics. Participants received training regarding informed consent for photography, privacy protection, image ownership, and the avoidance of identifiable photographs unless explicit permission had been obtained. Additional written consent was secured for photographs selected for dissemination, publication, or exhibition. All data were anonymised prior to analysis and reporting.

Given the potentially sensitive nature of family caregiving experiences, particular attention was paid to participant wellbeing throughout the study. Participants were informed that they could decline to answer questions, withdraw photographs from analysis, or discontinue participation at any stage without consequence. Where discussions elicited emotional discomfort, participants were offered opportunities for debriefing and referral to appropriate support services if required. These procedures align with established ethical principles for qualitative and participatory research, which emphasise respect for autonomy, minimisation of harm, and ongoing attention to participant wellbeing throughout the research process ( Liamputtong, 2020; Orb et al., 2001; Wang & Redwood-Jones, 2001).

### Data Collection

Data collection occurred over a twelve-week period and followed a structured nine-stage Photovoice process adapted from Wang and Burris (1997), Sutton-Brown (2014), and Pavlopoulou and Dimitriou (2020). The stages of data collection and participant engagement are summarised in Table 5.

**Table 5.** Photovoice timeline (9 Steps, 12 Weeks).

Step	Period	Description
Step 1	Week 1	Selection stage
Step 2	Week 2	Recruit participants, build contacts, and make introductions
Step 3	Week 3	Ethical photovoice training
Step 4	Week 4–5	Photo Capture and Visual Data Production
Step 5	Week 6	Photo-elicitation interviews, Categorization of themes
Step 6	Week 7	Group discussion with SHOWeD, Personal narrative & photo meaning
Step 7	Week 8–9	Co-analysis of photos & narratives, Grouping categories/themes, and critical analysis
Step 8	Week 10–11	Photo exhibitions in schools/communities, Sharing sessions with stakeholders, Documentation & publication of results
Step 9	Week 12	Evaluation of participation experience, action plan, and recommendations

The closing workshop allowed participants to reflect on the Photovoice process, discuss the learnings gained, and propose a plan for future advocacy. In total, the participants produced 47 photos, of which 18 were selected from 4 interviews and 2 FGDs, forming a primary data set for analysis.

The process began with participant orientation and relationship building, during which the study aims and ethical principles were discussed. Participants subsequently completed Photovoice training that focused on ethical photography, image selection, privacy protection, and reflective storytelling.

During weeks four and five, participants captured photographs documenting their everyday experiences of supporting a sibling with deafblindness. Participants were encouraged to photograph activities, environments, emotions, challenges, and sources of support that they considered meaningful to their caregiving experiences.

Individual photo-elicitation interviews were subsequently conducted, lasting between 45 and 60 minutes. Participants selected photographs that they considered most significant and discussed their meaning using the SHOWeD framework ( Wang & Burris, 1997): What do you See here? What is really Happening? How does this relate to Our lives? Why does this situation exist? What can we Do about it?

Two focus group discussions were then conducted, one involving rural participants and one involving urban participants. These discussions provided opportunities for collective reflection, comparison of experiences, and collaborative interpretation of photographs and narratives.

The final stages involved collaborative thematic grouping, community exhibitions within participating schools, and reflective evaluation sessions. Across the study, participants generated 47 photographs, from which 18 photographs were selected for detailed discussion and analysis. Data collection was intentionally iterative and participatory. Insights generated during interviews informed subsequent focus group discussions, while collaborative reflection sessions enabled participants to refine interpretations and identify issues they considered most significant. This iterative process enhanced the depth of data generated and ensured that participants remained active contributors throughout the research process rather than solely sources of data.

### Data Analysis

**Figure 1.**
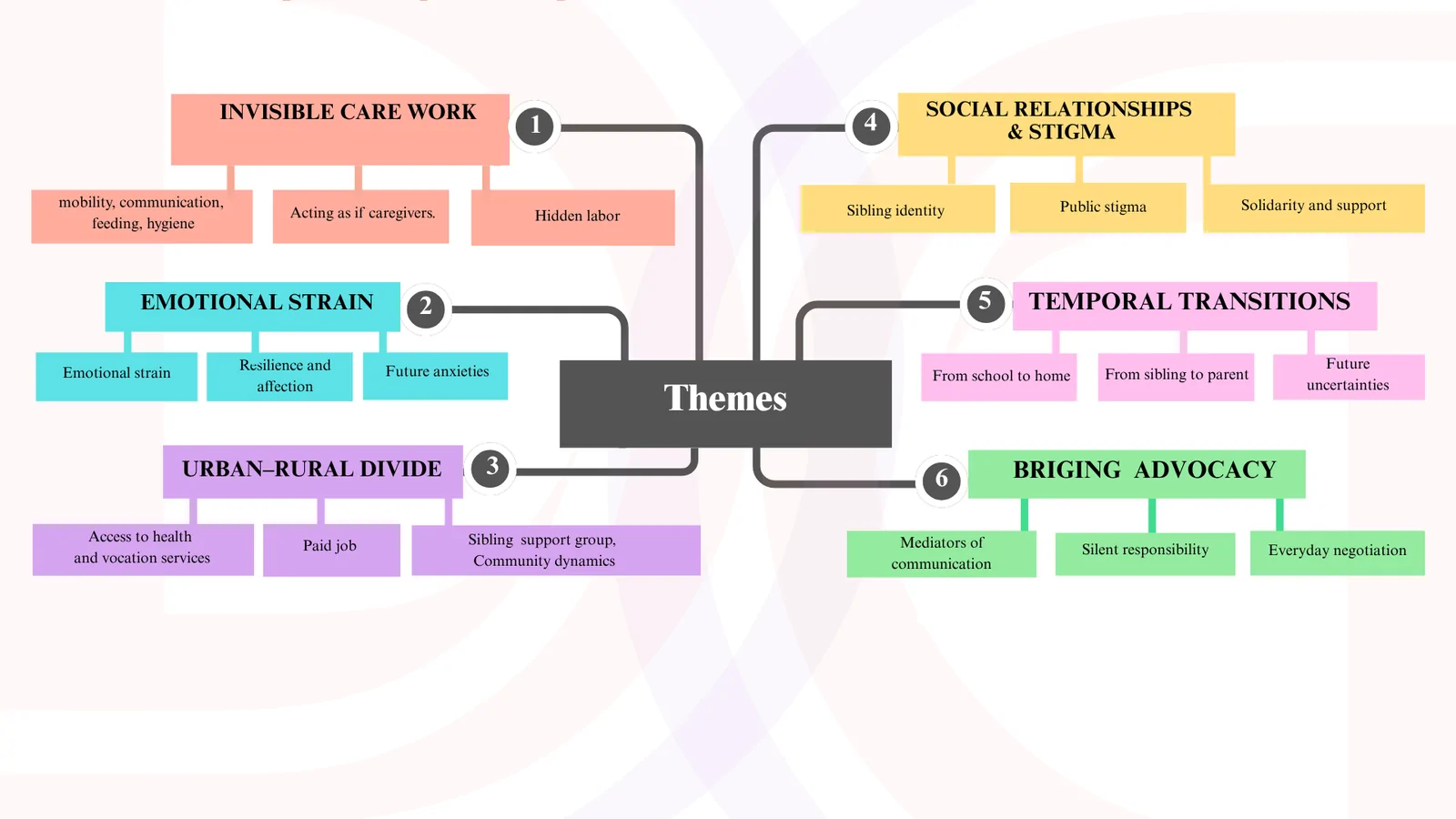
Thematic analysis results.

Data were analysed using reflexive thematic analysis informed by the work of Braun and Clarke (2006, 2021). The process of theme development and analytic progression is illustrated in Figure 1.

Analysis incorporated both visual and textual data, including photographs, interview transcripts, focus group discussions, field notes, and participant reflections.

The analysis involved six iterative phases. First, researchers familiarised themselves with the dataset through repeated reading of transcripts and examination of photographs. Second, initial codes were developed across both visual and narrative data. Third, codes were organised into preliminary thematic patterns. Fourth, themes were reviewed and refined through comparison with the entire dataset. Fifth, themes were defined and named to ensure conceptual clarity and coherence. Finally, themes were synthesised into an interpretive narrative that integrated participant quotations, visual data, and contextual insights.

Consistent with reflexive thematic analysis, themes were not understood to passively emerge from the data but were actively constructed through sustained engagement between researchers, participants, and the dataset ( Braun & Clarke, 2021). Analysis was therefore viewed as an interpretive and reflexive process rather than a purely descriptive exercise. Participant reflections generated during focus group discussions, collaborative interpretation sessions, and community exhibitions contributed to the ongoing refinement of codes, thematic patterns, and interpretive insights. The integration of visual and textual data enabled a richer understanding of caregiving experiences by capturing not only what participants described but also how they represented and made meaning of their everyday realities.

### Trustworthiness

Trustworthiness was established through strategies addressing credibility, dependability, confirmability, and transferability in accordance with Lincoln and Guba’s (1985) framework. Given the participatory nature of the study, trustworthiness was viewed as an ongoing process embedded throughout data generation, analysis, and interpretation rather than as a series of post-hoc verification procedures.

Credibility was enhanced through methodological triangulation involving photographs, interviews, focus group discussions, field notes, and participant reflections ( Denzin, 1978; Patton, 1999). Collaborative interpretation sessions enabled participants to contribute to the refinement of emerging themes, thereby strengthening the authenticity and resonance of findings, consistent with participatory and Photovoice approaches ( Wang & Burris, 1997; Catalani & Minkler, 2010). Dependability was supported through the maintenance of a comprehensive audit trail documenting methodological decisions, coding development, analytic discussions, and reflexive observations ( Lincoln & Guba, 1985). Confirmability was enhanced through reflexive memo writing, collaborative analysis among researchers, and ongoing critical examination of assumptions and interpretive decisions ( Lincoln & Guba, 1985; Nowell et al., 2017). Transferability was facilitated through the provision of rich descriptions of participant characteristics, family circumstances, geographical contexts, and caregiving environments, enabling readers to assess the applicability of findings to other settings and populations ( Lincoln & Guba, 1985).

Rather than seeking statistical generalisability, the study aimed to provide rich contextual insights into sibling caregiving experiences during post-school transition within Indonesian deafblindness contexts.

### Findings

Analysis of photographs, interviews, focus group discussions, and participant reflections generated four interconnected themes that illuminate how siblings understood, negotiated, and responded to caregiving responsibilities during the post-school transition of adolescents with congenital deafblindness and additional intellectual and mental disabilities. Although each theme is presented separately for analytic purposes, participants’ experiences were highly interconnected and reflected the dynamic interplay between family relationships, cultural expectations, geographical contexts, and disability-related support needs. The four themes were: (1) Invisible Caregiving and Role Transformation; (2) Emotional Burden, Resilience, and Uncertain Futures; (3) Geography, Stigma, and Unequal Support Systems; and (4) Siblings as Communication Brokers and Hidden Advocates.

Across all themes, caregiving was described not simply as a set of practical tasks but as an evolving relational and emotional process that shaped participants’ identities, aspirations, family relationships, and understandings of responsibility. While experiences differed between rural and urban settings, siblings consistently occupied positions of care, advocacy, and emotional support that were frequently overlooked within formal transition planning and disability support systems.

### Theme 1: Invisible Caregiving and Role Transformation

The first theme captures how siblings gradually assumed extensive caregiving responsibilities that extended far beyond conventional sibling relationships. Participants described caregiving not as a discrete set of tasks but as an ongoing and often invisible form of labour embedded within everyday family life. Their responsibilities encompassed personal care, communication support, supervision, household management, emotional reassurance, and advocacy. Although these activities were essential to the wellbeing and daily functioning of their siblings, they were frequently normalised within family environments and rarely recognised by schools, services, or wider society.

Photographs depicting household objects, hygiene routines, communication aids, and domestic spaces illustrated how caregiving became woven into participants’ everyday lives. Rather than perceiving caregiving as an exceptional responsibility, many participants described it as an expected component of family membership. However, this normalisation often obscured the time, effort, and emotional labour involved, rendering their contributions largely invisible despite their central role in supporting adolescents with deafblindness.

**Figure 2.**
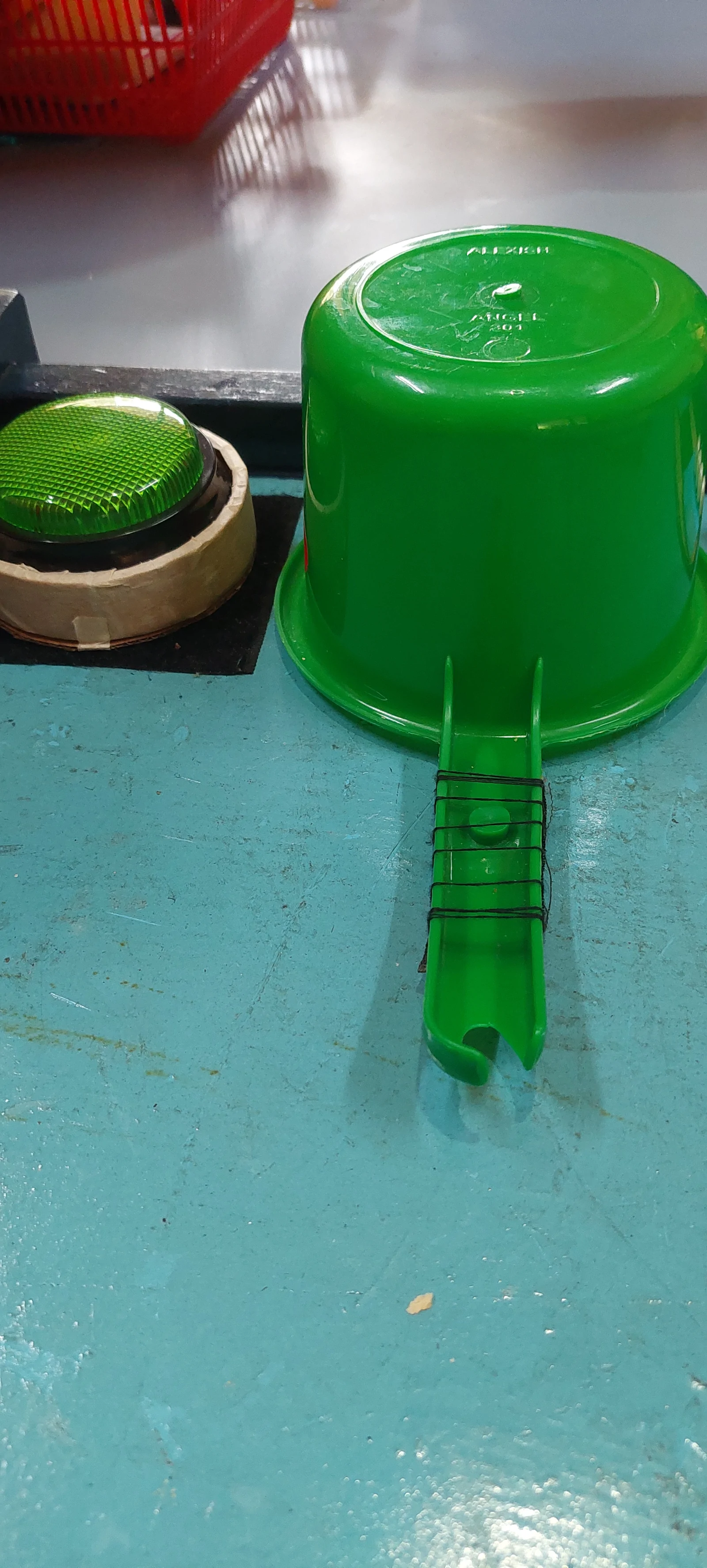
Illustrates a bathing dipper and communication button, symbolising the practical realities of caregiving.

Participants described how communication challenges frequently complicated routine activities and contributed to misunderstandings, frustration, and emotional exhaustion.

*“I don’t understand how to help my brother, because often he suddenly throws tantrums and hits when I’m in company. I can’t understand what he wants because I don’t understand how to communicate with him.”* (Participant 3).

This quotation illustrates how communication difficulties extended beyond practical interaction and frequently shaped emotional experiences of caregiving. Participants often described uncertainty regarding how best to interpret behaviours, needs, and emotional expressions, resulting in feelings of frustration, helplessness, and self-doubt. Communication therefore emerged not only as a functional challenge but also as a significant emotional component of sibling caregiving.

Although caregiving responsibilities were often viewed within families as normal or expected, participants described the cumulative impact of these duties on their personal lives. Several reported sacrificing leisure activities, educational opportunities, and social experiences in order to support their sibling.

*“Honestly, I feel like I’ve lost my playing time because of the non-stop but invisible domestic work.”* (Participant 5).

Importantly, caregiving responsibilities intensified during the transition from school to adulthood. Participants described a gradual but profound shift in family expectations following the reduction of school-based support and structured educational services. As opportunities for external assistance diminished, siblings increasingly assumed responsibilities that had previously been shared by teachers, therapists, support workers, or educational systems.

*“At school, there are teachers and friends who help. But once I got home, I had to stay with him.”* (Participant 1).

This transition represented more than a practical increase in caregiving tasks; it reflected a broader transformation of sibling identity and family role. Participants described moving from being primarily brothers and sisters to becoming quasi-caregivers, advocates, supervisors, and future sources of long-term support. The findings therefore suggest that post-school transition constitutes a dual transition: one experienced by adolescents with deafblindness as they move towards adulthood and another experienced by siblings as they negotiate evolving caregiving responsibilities and future expectations.

### Theme 2: Emotional Burden, Resilience, and Uncertain Futures

The second theme captures the emotional complexity of sibling caregiving during post-school transition. Participants described caregiving as simultaneously characterised by love, commitment, responsibility, exhaustion, anxiety, and uncertainty. Rather than presenting caregiving as either a burden or a source of fulfilment, siblings articulated emotionally ambivalent experiences in which affection and obligation coexisted with fatigue, frustration, and concern for the future. These emotional experiences were closely intertwined with participants’ identities, relationships, and expectations for adult life.

Many participants described feeling emotionally responsible for the safety and wellbeing of their sibling. This responsibility was particularly evident in public settings, where concerns about communication difficulties, behavioural challenges, or vulnerability increased their sense of vigilance.

*“It’s very tiring because everything has to be accompanied, but I love him very much.”* (Participant 4).

Participants frequently described a heightened sense of responsibility for their sibling’s safety, wellbeing, and social inclusion. This responsibility often extended beyond practical caregiving tasks and involved ongoing emotional vigilance, particularly in public environments where communication difficulties or behavioural challenges increased concerns regarding vulnerability and stigma. As a result, caregiving was experienced not only as physical labour but also as a sustained form of emotional labour that required constant attention and responsiveness.

*“Arriving home, I couldn’t rest because I still accompanied my sister, alternating with my mother. I don’t even have time alone.”* (Participant 3).

For several participants, caregiving responsibilities created tensions between family obligations and personal aspirations. Urban participants in particular described negotiating competing demands associated with education, employment, social relationships, and caregiving. Feelings of guilt frequently emerged when participants prioritised their own needs, suggesting that caregiving responsibilities shaped not only daily routines but also decisions regarding personal development and future opportunities.

**Figure 3.**
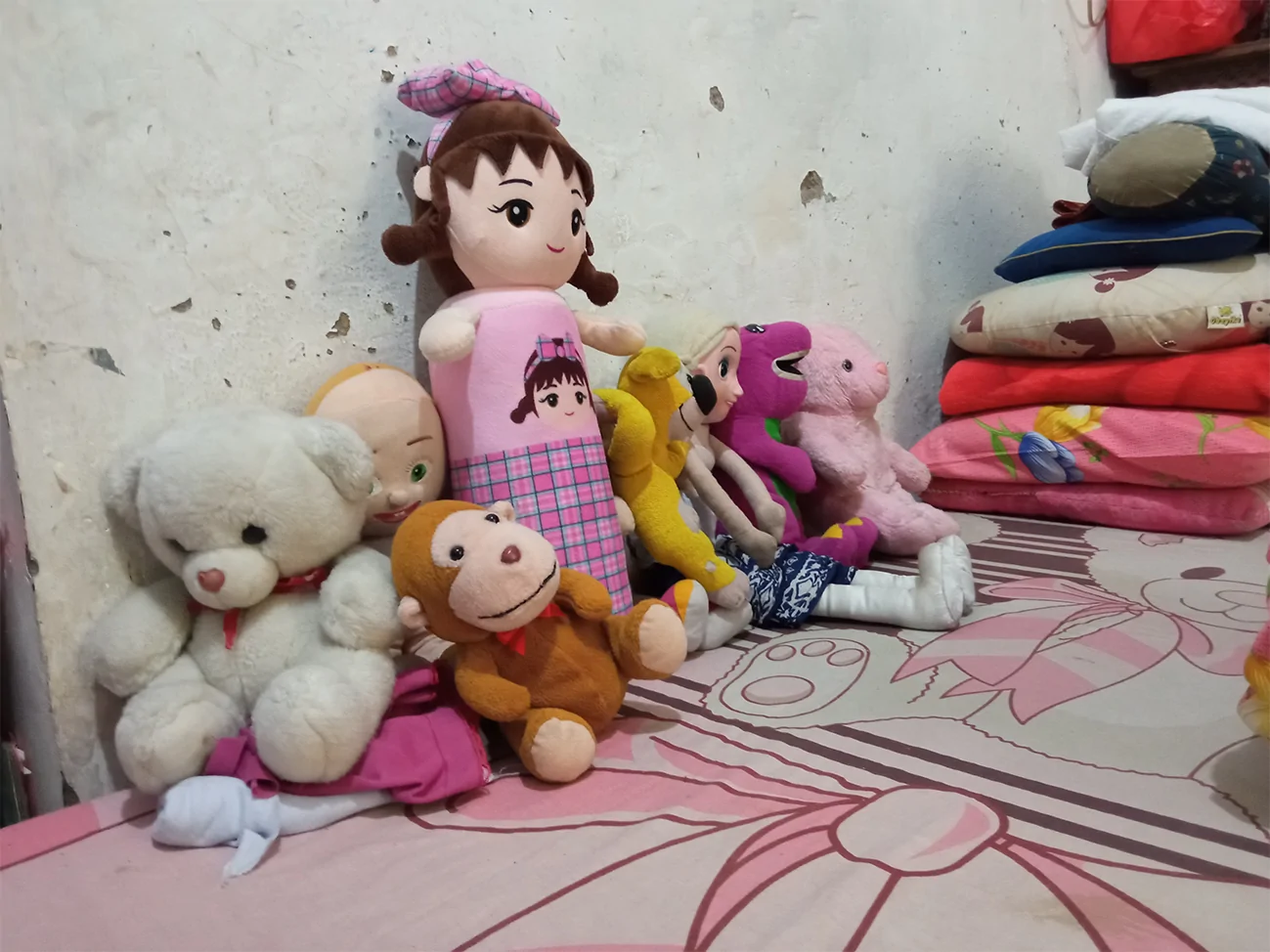
Dolls arranged together on a mattress, symbolising sibling attachment, resilience, and enduring family bonds.

At the same time, participants demonstrated considerable resilience. Figure 3, depicting dolls arranged together on a mattress, was interpreted by one participant as symbolising enduring sibling bonds, mutual dependence, and emotional closeness developed over many years.

Resilience was not presented as the absence of hardship but rather as an ongoing process of adaptation and meaning-making. Participants frequently acknowledged emotional strain while simultaneously describing caregiving as an expression of familial commitment, reciprocity, and enduring sibling bonds. Their narratives suggest that resilience emerged through the continual negotiation of challenges rather than through the elimination of adversity.

Future uncertainty emerged as a particularly powerful concern. Participants worried about educational opportunities, employment prospects, marriage, and the long-term care of their sibling following parental ageing or death.

*“I love my sister, but sometimes I am tired and confused too. I don’t know if my marriage will be good because in the future I will definitely bring her into my little family.”* (Participant 1).

Future uncertainty emerged as one of the most significant emotional concerns described by participants. Unlike many adolescents and young adults whose future planning primarily centred on education, employment, or family formation, participants frequently viewed these aspirations through the lens of anticipated caregiving responsibilities. Concerns regarding parental ageing, future care arrangements, financial security, marriage, and long-term support were common. Consequently, caregiving responsibilities influenced not only participants’ present experiences but also their imagined futures and life trajectories.

Collectively, these findings suggest that sibling caregiving extends beyond immediate practical support and becomes embedded within broader processes of identity formation, future planning, and family responsibility. The emotional consequences of caregiving were therefore experienced not only in the present but also through participants’ anticipations of future roles, relationships, and obligations.

### Theme 3: Geography, Stigma, and Unequal Support Systems

The third theme illustrates how sibling caregiving was shaped not only by disability-related needs but also by broader geographical, social, and structural contexts. Participants consistently described caregiving as occurring within environments characterised by differing levels of service availability, community support, accessibility, and social acceptance. Although siblings across rural and urban settings assumed similar caregiving responsibilities, the resources available to support these roles varied considerably, influencing both caregiving experiences and perceptions of future opportunities.

Urban participants generally reported greater access to specialist services, educational programmes, and support organisations. However, these advantages were frequently offset by financial pressures, transportation difficulties, bureaucratic barriers, and reduced community cohesion.

**Figure 4.**
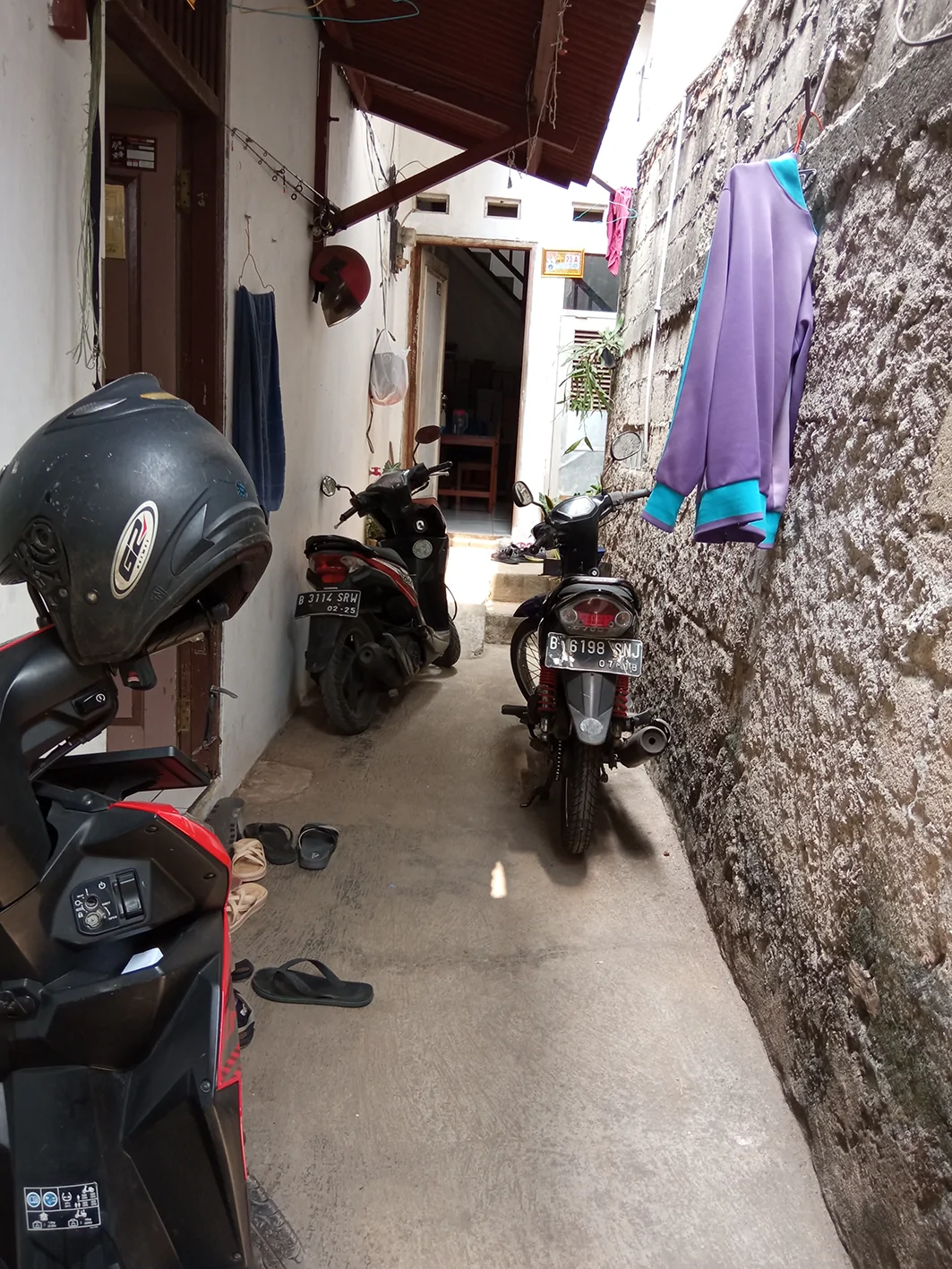
Narrow alleyway leading to a family home, illustrating environmental barriers to healthcare and emergency access.

Participants used this image to demonstrate how environmental barriers could complicate access to healthcare and emergency services.

*“Once I had to rent a stretcher and four people to take my little brother to the big street to access the ambulance because we lived in a narrow and crowded alley.”* (Participant 3).

While urban environments were often perceived as offering greater access to specialist services and educational opportunities, participants emphasised that service availability did not necessarily equate to accessibility. Financial constraints, transportation difficulties, bureaucratic processes, and complex urban environments frequently limited the practical benefits of available services. Consequently, participants often experienced a disconnect between the theoretical availability of support and their ability to access it in everyday life.

Rural participants described a contrasting experience characterised by limited formal support but stronger informal networks. Although access to specialist healthcare, educational services, and disability programmes was often restricted, participants frequently reported receiving practical assistance, emotional support, and understanding from extended family members and local communities. These findings suggest that kinship networks functioned as an important source of resilience, partially compensating for deficiencies within formal support systems.

Across both settings, disability stigma remained a significant challenge. Participants reported experiences of social exclusion, embarrassment, misunderstanding, and judgment from others.

*“When I brought my brother, I was often reprimanded because it is troublesome and noisy if he has a tantrum. I feel embarrassed and guilty.”* (Participant 6).

Experiences of stigma extended beyond the individual with deafblindness and frequently affected siblings themselves. Participants described feelings of embarrassment, guilt, frustration, and social isolation arising from negative public reactions to disability-related behaviours. Such experiences reflect processes of secondary or courtesy stigma, whereby family members experience social consequences associated with their relationship to a person with a stigmatised condition. Stigma therefore emerged not only as a social challenge for adolescents with deafblindness but also as a factor shaping sibling identity, emotional wellbeing, and participation in community life.

Collectively, these findings reveal a geographical paradox in which urban environments offered greater service availability but weaker social connectedness, whereas rural communities provided stronger interpersonal support despite fewer formal resources. The experiences of participants highlight the importance of considering disability support as both a structural and relational phenomenon, shaped not only by the presence of services but also by the quality of social relationships, community inclusion, and cultural understandings of disability.

### Theme 4: Siblings as Communication Brokers and Hidden Advocates

The fourth theme captures the ways in which siblings acted as communication brokers, advocates, and facilitators of social inclusion across family, community, and service contexts. Beyond providing practical and emotional support, participants frequently assumed responsibility for helping others understand the needs, preferences, and experiences of their sibling with deafblindness. In doing so, they occupied a critical intermediary position between adolescents with deafblindness and the wider social world.

In healthcare settings, siblings often served as communication brokers, helping professionals understand the needs of their brother or sister.

*“If I go to the doctor, I will talk because parents sometimes lack confidence and stutter.”* (Participant 4).

Participants frequently described translating, interpreting, and clarifying communication during interactions with healthcare professionals, educators, and community members. In many cases, siblings compensated for communication barriers within systems that were not adequately equipped to accommodate the needs of individuals with deafblindness. Their accounts suggest that communication brokerage functioned as an essential but largely unrecognised form of support that facilitated access to services, information, and participation.

Participants also described advocating within schools, communities, and social settings. Their role extended beyond caregiving to include educating others about deafblindness, promoting inclusion, and creating opportunities for participation.

**Figure 5.**
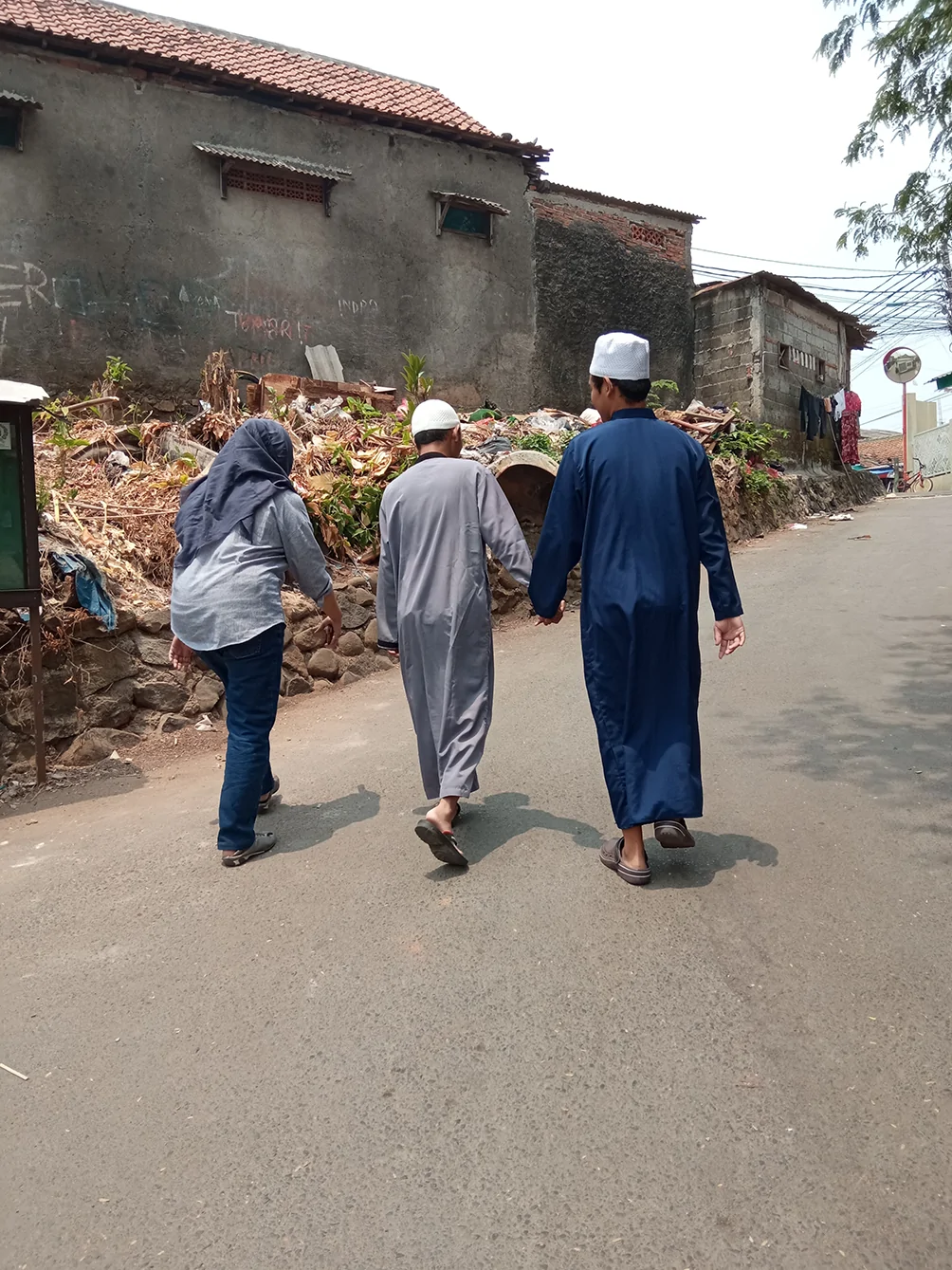
Siblings accompanying a deafblind adolescent to the mosque, illustrating everyday advocacy and community inclusion.

Figure 5, which depicts siblings accompanying a deafblind adolescent to a mosque, reflected how advocacy often occurred through everyday acts of inclusion. Participants described making deliberate efforts to ensure their sibling remained visible within community life despite social barriers.

*“Even though I wasn’t invited, I always invited my deafblind brother to join the Independence Day celebrations in the village. People often looked at him strangely, but that was my chance to explain about my brother.”* (Participant 6).

Advocacy frequently occurred through everyday actions rather than formal roles or organised activities. Participants described actively challenging misconceptions, promoting understanding, and creating opportunities for inclusion within schools, neighbourhoods, religious settings, and community events. These efforts reflected a commitment not only to supporting their sibling but also to fostering greater social acceptance and participation within their communities.

Importantly, advocacy was rarely recognised as a formal responsibility. Participants generally viewed these activities as a natural extension of sibling relationships despite the considerable emotional, social, and practical labour involved. Their narratives suggest that siblings functioned as hidden advocates whose contributions were essential to communication, service access, social participation, and community inclusion. In many instances, siblings appeared to bridge gaps within formal support systems by undertaking advocacy roles that might otherwise be performed by specialised professionals or dedicated support services. The findings therefore highlight the central yet frequently invisible role of siblings in enabling participation and citizenship for adolescents with deafblindness during the transition to adulthood.

## Discussion

This study explored the experiences of siblings supporting adolescents with congenital deafblindness and additional intellectual and mental disabilities during post-school transition in rural and urban Indonesia. Using a participatory Photovoice methodology, the findings demonstrate that siblings occupy complex and frequently unrecognised roles as caregivers, advocates, communication brokers, and sources of emotional support within family systems. While previous deafblindness research has largely prioritised parental perspectives ( Dammeyer, 2014; Janssen et al., 2003), the present study highlights siblings as central actors within transition processes whose contributions are critical to the wellbeing, participation, and future support of adolescents with deafblindness. By foregrounding sibling perspectives within a low-resource and family-centred context, the study contributes new insights into how disability, transition, family relationships, and caregiving intersect across the life course. In doing so, the study responds to calls for greater attention to the experiences of siblings of individuals with complex disabilities, whose perspectives remain underrepresented in transition and deafblindness research despite their increasingly important roles in long-term support networks ( Heller & Arnold, 2010; Orsmond & Seltzer, 2007).

The findings contribute to a growing body of literature recognising that transition to adulthood is not solely an individual developmental process but a family transition involving interconnected changes in roles, responsibilities, identities, and support systems ( Francis et al., 2018; Trainor et al., 2020). Importantly, this study suggests that post-school transition should also be understood as a transition in sibling identity. As structured educational supports diminish and family responsibilities increase, siblings frequently move from primarily relational roles towards more sustained caregiving, advocacy, and supervisory responsibilities. This finding extends existing transition literature by demonstrating how the movement from school-based support to family-based care reshapes sibling identities, future expectations, and life trajectories in enduring ways. Consistent with life-course perspectives on disability and family caregiving, siblings’ experiences were shaped not only by present responsibilities but also by anticipated future obligations, including concerns about long-term care, financial security, and family continuity ( Elder, 1998; Seltzer et al., 2005). These findings suggest that transition planning should recognise siblings as key stakeholders whose needs, aspirations, and preparedness influence the sustainability of support arrangements across adulthood.

Taken together, the findings suggest that sibling caregiving during post-school transition should be understood as a multidimensional process involving invisible labour, emotional ambivalence, geographical inequality, and hidden advocacy. The following sections discuss these contributions in relation to existing literature, theory, and implications for policy and practice.

### Invisible Caregiving and the Hidden Workforce of Disability Support

A central finding of this study was the largely invisible nature of sibling caregiving. Participants described providing extensive support with personal care, communication, supervision, emotional regulation, household management, and community participation. These responsibilities were often embedded within everyday family life and consequently remained unrecognised by formal support systems. The normalisation of caregiving within family contexts frequently obscured the scale and complexity of siblings’ contributions. As a result, participants often undertook substantial practical and emotional responsibilities without formal recognition, support, or preparation. This finding suggests that siblings constitute an important but largely invisible workforce within disability support systems, particularly in contexts where family-based care remains the primary source of assistance.

The concept of invisible care work offers a useful framework for understanding these experiences. Previous studies have demonstrated that family caregiving frequently involves substantial unpaid labour that remains hidden from policymakers, professionals, and wider society ( Bigby et al., 2015; Hall & Rossetti, 2018). The present findings extend this literature by illustrating how sibling caregiving within deafblindness contexts represents a distinctive form of invisible labour characterised by communication mediation, constant supervision, and long-term responsibility.

Importantly, participants rarely described caregiving as a voluntary choice. Instead, caregiving emerged as a relational obligation shaped by family expectations, emotional attachment, and cultural values. This finding is consistent with Family Systems Theory, which proposes that changes affecting one family member influence the functioning and adaptation of the wider family system ( Bowen, 1978; Turnbull et al., 2015). Within this perspective, disability is not experienced solely by the individual but by the family as a relational unit, with family members adapting their roles and responsibilities in response to changing support needs ( Kyzar et al., 2012; Turnbull et al., 2015). Siblings therefore become integral contributors to family adaptation, continuity, and resilience, particularly during periods of transition when support needs to intensify. This interpretation is further supported by research demonstrating that siblings of individuals with disabilities frequently assume caregiving, advocacy, and emotional support roles that evolve across the life course and become increasingly prominent during the transition to adulthood ( Heller & Arnold, 2010; Lee et al., 2021; Francis et al., 2018). In contexts where formal services are limited and family-based care remains the primary source of support, these responsibilities may be especially pronounced, reinforcing the central role of siblings within family support networks ( Meyer et al., 2011; Trainor et al., 2020).

### Emotional Ambivalence, Resilience, and Future Planning

The emotional experiences described by participants reveal the complexity of sibling caregiving during post-school transition. Consistent with previous studies involving siblings of individuals with disabilities ( Arcous et al., 2024; Giallo et al., 2012), participants simultaneously expressed affection, commitment, frustration, anxiety, exhaustion, and hope. Rather than representing contradictory experiences, these emotions appeared deeply interconnected and reflected the relational nature of caregiving. The findings suggest that sibling caregiving is characterised by emotional ambivalence, whereby positive and challenging experiences coexist and are continually negotiated within everyday family life.

The findings further suggest that resilience should not be understood as the absence of hardship or emotional distress. Instead, resilience emerged as a dynamic process of adaptation, meaning-making, and ongoing negotiation in response to changing caregiving demands. Participants demonstrated considerable adaptability despite limited formal support, communication challenges, and uncertainty regarding future responsibilities. This finding aligns with contemporary understandings of resilience as a relational and contextual process shaped by family relationships, social resources, and lived experience rather than as an individual trait or fixed characteristic. These findings are also consistent with family resilience perspectives, which conceptualise resilience as a dynamic process through which families adapt, reorganise, and maintain functioning in the face of adversity rather than as an individual characteristic ( Walsh, 2016). Participants’ accounts suggest that resilience emerged through ongoing negotiation of caregiving responsibilities, emotional challenges, and future uncertainties while maintaining strong familial commitments and relationships.

One of the most significant findings was the prominence of future-oriented concerns. Participants frequently reflected on how caregiving responsibilities might influence educational opportunities, employment trajectories, intimate relationships, marriage, and future family life. Such concerns mirror findings from international disability research, where siblings often report anxiety regarding long-term caregiving responsibilities following parental ageing, declining health, or death ( Heller & Arnold, 2010; Strohm, 2018). However, the present study suggests that these concerns are not limited to future caregiving arrangements alone. Rather, anticipated responsibilities were already shaping participants’ current decision-making, aspirations, and perceptions of adulthood, indicating that caregiving influences both present experiences and imagined futures. The findings therefore highlight the emergence of what may be understood as anticipatory caregiving, whereby future expectations of care begin to influence life choices long before formal caregiving responsibilities are fully assumed.

The prominence of future-oriented concerns suggests that transition planning should extend beyond adolescents with disabilities to include siblings who may become future caregivers, advocates, and decision-makers. Current transition programmes frequently focus on the individual with a disability and their parents, overlooking siblings despite their likely long-term involvement in care and support. Greater recognition of sibling perspectives within transition planning may therefore facilitate more sustainable family-centred approaches to adulthood and long-term care. Supporting siblings through information provision, future planning discussions, and opportunities for participation in decision-making may help strengthen both family resilience and long-term transition outcomes.

### Geography, Social Context, and Unequal Transition Experiences

An important contribution of this study is the demonstration that sibling experiences are shaped by geography as well as disability. The comparison between rural and urban contexts revealed that transition experiences are embedded within broader social, economic, cultural, and environmental conditions rather than being determined solely by individual or family characteristics.

Urban participants generally reported greater access to specialist services and educational opportunities but also described social fragmentation, economic pressures, transportation challenges, and limited community support. Rural participants experienced fewer formal services but often reported stronger kinship networks and community familiarity. These findings can be understood through Bronfenbrenner’s Ecological Systems Theory, which emphasises the reciprocal influence of individual, family, community, institutional, and societal environments on development and wellbeing ( Bronfenbrenner, 1979). The findings suggest that sibling caregiving was shaped not only by disability-related needs but also by the wider ecological systems within which families lived.

The findings further suggest that service availability alone does not necessarily equate to meaningful support. While urban participants had greater access to services, they frequently described feelings of isolation and burden. Conversely, some rural participants identified social connectedness, kinship solidarity, and collective responsibility as important sources of resilience despite significant resource limitations. This observation highlights the importance of considering both formal and informal support systems within transition planning and reinforces the need to understand disability support as both a structural and relational phenomenon.

Disability stigma emerged as a persistent challenge across both settings. Participants described experiences of social judgment, exclusion, embarrassment, and misunderstanding. Consistent with previous deafblindness research ( Simcock, 2014, 2017), stigma functioned not only as a barrier to participation for adolescents with deafblindness but also affected siblings through processes of secondary or courtesy stigma and social isolation. These findings suggest that stigma extends beyond the individual with a disability and can influence family relationships, emotional wellbeing, and community participation. Greater public awareness, community education, and inclusive practices are therefore required to support not only individuals with deafblindness but also the family members who contribute to their everyday care and social inclusion.

Collectively, these findings reveal a geographical paradox in which urban environments offered greater service availability but weaker social connectedness, whereas rural communities provided stronger interpersonal support despite fewer formal resources. The study therefore highlights the importance of understanding transition experiences as products of interacting ecological systems, social relationships, and structural inequalities rather than solely as individual or family-level challenges.

### Siblings as Communication Brokers and Hidden Advocates

One of the most novel findings of this study concerns the advocacy and communication roles undertaken by siblings. Participants frequently acted as communication brokers between adolescents with deafblindness, family members, professionals, schools, healthcare services, and community organisations. In doing so, they occupied a critical intermediary position that extended well beyond traditional sibling relationships. Rather than simply providing support within the family, siblings actively facilitated communication, participation, understanding, and access across multiple social and institutional settings.

The advocacy roles identified in this study resonate with previous work describing siblings as protectors, interpreters, educators, and future planners ( Burke et al., 2015; Hall & Rossetti, 2018). However, the present findings suggest that advocacy within deafblindness contexts may be particularly intensive due to the communication barriers associated with combined sensory impairment and additional intellectual and mental disabilities. Siblings frequently translated needs, interpreted behaviours, clarified communication, and supported interactions with services that were not always equipped to accommodate the complex communication requirements of individuals with deafblindness.

Importantly, participants rarely viewed advocacy as a formal responsibility. Instead, advocacy emerged as a natural extension of sibling relationships and family commitment. Through everyday actions, participants challenged stigma, promoted inclusion, facilitated access to services, and enabled participation in community life. These findings suggest that siblings function as hidden advocates whose contributions remain largely absent from policy frameworks, transition planning models, and disability support systems despite their central role in promoting communication, social participation, and access to opportunities.

The findings further suggest that siblings frequently compensate for gaps within formal support systems by undertaking advocacy roles that might otherwise be performed by specialised professionals, support workers, or dedicated service providers. Consequently, advocacy should be recognised not simply as an individual or family activity but as an important component of broader disability support ecosystems. Greater recognition of sibling advocacy may therefore contribute to more inclusive and sustainable approaches to transition planning and long-term support.

From a family systems perspective, these advocacy activities represent an important mechanism through which families adapt to disability-related challenges and maintain participation within wider social systems ( Bowen, 1978).

Recognising siblings as key stakeholders within disability support systems represents an important step towards more family-centred approaches to transition planning. Schools, healthcare providers, and social service agencies should consider how siblings can be meaningfully included in transition discussions, future planning processes, and service coordination activities while also ensuring that appropriate emotional, informational, and practical support is available to meet their own needs.

### Methodological Contribution

The study also contributes methodologically to disability, family, and deafblindness research. While previous studies exploring family experiences of deafblindness have predominantly relied upon interviews, surveys, or parental accounts, the use of Photovoice enabled participants to document, interpret, and critically reflect upon their own experiences through visual and narrative means. The photographs provided rich representations of caregiving practices, emotional experiences, environmental barriers, communication challenges, and advocacy activities that may have remained difficult to articulate through conventional interview methods alone.

Consistent with participatory disability research principles, Photovoice facilitated the active involvement of participants in knowledge production rather than positioning them solely as subjects of research ( Wang & Burris, 1997). Through the processes of image creation, reflection, discussion, and collaborative interpretation, participants were able to identify issues of personal significance and contribute directly to the development of research insights. This participatory approach aligns with contemporary calls for greater inclusion of disabled individuals and family members in the production of knowledge concerning their own lives and experiences ( Cluley, 2016; Pavlopoulou & Dimitriou, 2020).

Importantly, the methodology enabled the visualisation of caregiving experiences that are frequently hidden within private family environments. Photographs captured not only practical caregiving activities but also emotional relationships, environmental constraints, social participation, and everyday acts of advocacy. The integration of visual and textual data therefore provided a more nuanced and contextualised understanding of sibling caregiving than might have been achieved through interviews alone.

The study further demonstrates the value of Photovoice for exploring the experiences of groups whose perspectives have historically been underrepresented within disability research. By foregrounding sibling voices and supporting participant-led interpretation, the methodology generated insights into caregiving, transition, and family life that may otherwise have remained obscured. As such, the findings contribute not only to substantive knowledge regarding deafblindness and sibling caregiving but also to the ongoing development of participatory and inclusive approaches within disability research.

### Implications for Policy, Practice, and Research

The findings have important implications for policy, practice, and future research. First, transition planning should move beyond an individualised focus on the adolescent with a disability and adopt a broader family-centred approach that recognises the interconnected roles of parents, siblings, and wider support networks. The findings demonstrate that siblings frequently assume substantial caregiving, advocacy, communication, and emotional support responsibilities during post-school transition, yet their contributions remain largely invisible within policy frameworks and service provision.

Second, educational, healthcare, and social service professionals should actively recognise siblings as key stakeholders within transition processes. Opportunities for information sharing, future planning, skills development, and emotional support should be made available to siblings alongside parents and primary caregivers. Greater involvement of siblings in transition planning may facilitate more sustainable long-term support arrangements and improve continuity of care across the transition to adulthood.

Third, the findings highlight the importance of addressing geographical inequalities in disability support. Policymakers and service providers should consider how both formal services and informal community networks contribute to transition outcomes. While increasing access to specialist services remains important, strengthening community inclusion, social connectedness, and family support mechanisms may be equally critical for promoting positive transition experiences.

The study also highlights the need for greater recognition of sibling advocacy within disability policy and practice. Siblings frequently acted as communication brokers and informal advocates, supporting access to services, facilitating participation, and promoting inclusion. Formal recognition of these roles may help ensure that siblings receive appropriate support while also strengthening family-centred approaches to disability care.

Future research should examine sibling experiences longitudinally to better understand how caregiving, advocacy, and support roles evolve across the life course. Comparative studies across different cultural, socioeconomic, and service contexts may further enhance understanding of how disability, family relationships, and transition intersect. Additional research exploring gender, socioeconomic status, family structure, and the experiences of siblings across different disability groups may provide valuable insights into factors influencing caregiving trajectories, wellbeing, and long-term outcomes. Research evaluating sibling-inclusive transition interventions would also represent an important next step in translating evidence into practice.

### Strengths and Limitations

This study contributes to the limited body of literature examining sibling experiences within deafblindness and post-school transition contexts. Several strengths warrant consideration. First, the study addresses an important gap in deafblindness research by foregrounding the perspectives of siblings, a group that has historically received considerably less attention than parents, educators, and healthcare professionals. Existing research on disability and family caregiving has increasingly recognised siblings as important contributors to lifelong support, advocacy, and family adaptation, yet their experiences remain underexplored, particularly within deafblindness contexts ( Meyer et al., 2011; Heller & Arnold, 2010). By focusing on siblings, this study provides novel insights into caregiving, advocacy, and transition experiences that are frequently overlooked despite their significance for long-term family functioning and future planning.

Second, the study employed a participatory Photovoice methodology that enabled participants to document, interpret, and critically reflect upon their experiences through both visual and narrative forms of expression. Photovoice has been widely recognised as an empowering participatory approach that facilitates the expression of lived experiences, particularly among groups whose perspectives are often marginalised or underrepresented in research ( Wang & Burris, 1997; Catalani & Minkler, 2010). The integration of photographs, individual interviews, focus group discussions, and collaborative reflection generated rich and contextualised data that may not have been accessible through conventional interview methods alone. This approach facilitated participant engagement and enabled the exploration of caregiving experiences that frequently remain hidden within private family environments.

Third, the inclusion of participants from both rural and urban regions of Indonesia enabled exploration of how geographical context influenced caregiving experiences, support systems, stigma, and access to services. This comparative perspective strengthened the study by illuminating the interaction between disability, family life, and broader social and structural environments. Such contextual understanding is particularly important in disability research, where access to resources and opportunities is often shaped by environmental and sociocultural factors ( World Health Organization, 2011).

Fourth, the study generated both substantive and methodological contributions. In addition to advancing understanding of sibling caregiving within deafblindness contexts, the findings demonstrate the value of participatory visual methodologies for exploring the experiences of groups whose voices are often underrepresented within disability research. The study therefore contributes to ongoing calls for more inclusive and participatory approaches that recognise people with lived experience and their families as active contributors to knowledge production ( Nind, 2014).

Several limitations should also be considered when interpreting the findings. The study involved a relatively small purposive sample of eight participants recruited through special education schools. Although the sample generated substantial depth of data and was appropriate for an intensive Photovoice study, the findings are not intended to be statistically representative of all siblings of adolescents with deafblindness in Indonesia or elsewhere. Consistent with qualitative inquiry, the aim was to generate in-depth and contextually situated understanding rather than statistical generalisation. Instead, the findings may offer analytical or theoretical insights that can inform understanding of similar contexts ( Lincoln & Guba, 1985).

Although the study included participants from rural and urban regions, the influence of gender, socioeconomic status, family structure, religion, and cultural expectations was not examined in depth. These factors appeared to shape caregiving experiences in important ways and warrant further investigation. Future studies may benefit from exploring how such intersecting influences contribute to variations in sibling roles, resilience, wellbeing, advocacy, and future planning. Such an approach would align with growing recognition of intersectionality as an important framework for understanding disability and family experiences across diverse social contexts ( Crenshaw, 1989; Shakespeare, 2018).

As with all qualitative research, findings were developed through interpretive engagement between participants and researchers. While reflexive practices, methodological triangulation, collaborative analysis, participant involvement, and an audit trail were employed to enhance trustworthiness, alternative interpretations remain possible. This should be viewed as a strength of qualitative inquiry rather than a methodological weakness, reflecting the complexity and context-dependent nature of lived experience ( Braun & Clarke, 2021).

Finally, the cross-sectional design captured sibling experiences at a particular point within the transition process. Transition to adulthood is dynamic and unfolds over time. Longitudinal research is therefore needed to examine how sibling responsibilities, relationships, resilience, advocacy roles, and future expectations evolve as adolescents with deafblindness move into adulthood and family circumstances change. Despite these limitations, the study provides one of the few in-depth explorations of sibling experiences within deafblindness transition contexts and offers important insights for research, policy, and practice.

### Conclusion and Implications

This study explored the experiences of siblings supporting adolescents with congenital deafblindness and additional intellectual and mental disabilities during post-school transition in rural and urban Indonesia. Using a participatory Photovoice methodology, the findings demonstrate that siblings occupy complex roles that extend beyond conventional family relationships, encompassing caregiving, communication support, advocacy, emotional regulation, and future planning. Despite their substantial contributions, these roles frequently remain unrecognised within disability services, transition planning processes, and policy frameworks.

A key contribution of this study is the identification of post-school transition as not only a developmental transition for adolescents with deafblindness but also a transition in sibling identity and responsibility. As educational supports diminish and family caregiving demands increase, siblings often assume increasingly significant roles within support networks. Across both rural and urban contexts, participants described experiences characterised by invisible caregiving, emotional ambivalence, resilience, advocacy, and uncertainty regarding future responsibilities. Although geographical context shaped access to services and social support, the central role of siblings within family support systems remained consistent.

By foregrounding sibling perspectives, this study extends current understandings of family-centred transition processes and challenges the parent-focused emphasis that dominates much of the existing literature. The findings also demonstrate the value of participatory visual methodologies for capturing caregiving experiences and family dynamics that may remain obscured through traditional research approaches.

The findings have important implications for policy and practice. Transition planning should adopt a genuinely family-centred approach that recognises siblings as active participants in support networks rather than peripheral family members. Educational, health, and social care professionals should provide opportunities for siblings to participate in transition discussions, access relevant information, and receive practical and emotional support. Disability policies and programmes should also recognise the significant contributions made by sibling caregivers and address geographical inequalities that influence access to support.

Future research should examine sibling experiences longitudinally to better understand how caregiving, advocacy, and support roles evolve across adulthood and changing family circumstances. Comparative studies across different cultural, socioeconomic, and service contexts would further strengthen understanding of how disability, family relationships, and transition intersect.

In conclusion, siblings represent a largely hidden yet essential component of support systems for adolescents with deafblindness. Their contributions extend beyond caregiving to encompass advocacy, communication, inclusion, and family continuity. Recognising, valuing, and supporting these roles is fundamental to developing more inclusive, equitable, and sustainable approaches to deafblindness care and transition support.
