# Supplementary material for: Unseen Care, Unequal Worlds: Photovoice Narratives of Siblings Supporting Deafblind Adolescents in Indonesia
Source: F1000Res. 2026 Jul 13;15:77. Originally published 2026 Jan 19. [Version 2] doi: 10.12688/f1000research.171949.2 (PMC13555031; doi:10.12688/f1000research.171949.2)
Supplement: Peer review history

# Reviewer report: Reviewer response for version 2

**DOI:** 10.5256/f1000research.203922.r501102

**Published:** 2026-09-24

**Authors:** Gro Hege Saltnes Urdal

**Affiliations:** 1 Western Norway University of Applied Sciences, Bergen,, Norway

The authors have made substantial improvements to the manuscript. The revised version now provides a more comprehensive and nuanced description of the research topic, methodology, and discussion, thereby strengthening its contribution to field. The authors have also done a good job of improving the overall structure of the article, making it easier for readers to follow the authors’ arguments and the progression of the analysis.

 There are, however, some minor changes that could have been made to improve the article even further. For example, there is some repetition in the latter part of the *Introduction*, particularly in the sections *Post-School Transition and Family Caregiving*, *Sibling Caregiving*, and *Invisible Labour and Family Relationships*. In particular, the discussion of siblings’ lifelong relationships with young people with deaf blindness and the support they provide is reiterated across several sections. Similar repetition is also evident in the *Discussion*. Although the *Discussion* is thoughtfully organized into subsections, several points are repeated across these sections. To some extent, this is understandable, as certain arguments are relevant to multiple themes. However, the arguments are often presented in very similar terms and with comparable levels of detail, resulting in a degree of repetition that may be excessive.

 In addition, the description of Photovoice would be more appropriately placed under the *Methodology* heading, as it is presented as a research method. Since the preceding sections focus on previous research, the manuscript would be more coherent if the *Research Gap*, *Aim*, and *Research Question* were presented before the description of Photovoice. Furthermore, Photovoice is described again within the *Methodology* section, where this information appears to fit more naturally. Relocating the earlier discussion of Photovoice to the *Methodology* section would improve the overall flow of the manuscript and help avoid unnecessary repetition.

 Finally, the overview of the thematic analysis presents six themes, whereas the Findings section appears to condense these into four themes without explanation. Providing a brief account of how and why this condensation occurred would enhance transparency and help readers better understand the analytical process.

 Nevertheless, the resubmission is approved.

Is the work clearly and accurately presented and does it cite the current literature?

Partly

If applicable, is the statistical analysis and its interpretation appropriate?

Partly

Are all the source data underlying the results available to ensure full reproducibility?

No

Is the study design appropriate and is the work technically sound?

Partly

Are the conclusions drawn adequately supported by the results?

Partly

Are sufficient details of methods and analysis provided to allow replication by others?

No

Reviewer Expertise:

Deafblindness, interpreting, qualitative research, participatory research

I confirm that I have read this submission and believe that I have an appropriate level of expertise to confirm that it is of an acceptable scientific standard.

# Reviewer report: Reviewer response for version 2

**DOI:** 10.5256/f1000research.203922.r501103

**Published:** 2026-08-26

**Authors:** Shirley Dumassais

**Affiliations:** 1 University of Montreal, Montreal, Québec, Canada

Thank you to the authors for the extensive improvements made to the manuscript. The overall quality has improved significantly.

**Minor comments:**

- The *gender* variable should use the terms *men/women* unless you are specifically referring to biological sex. If you are, please correct this. If sex is the variable of interest, please provide a rationale for its inclusion and clarify how this information was collected.
- The **Findings** heading appears misplaced and reads as though it is a subsection of the Methods section rather than a standalone section. Consider revising the formatting or section structure to improve clarity.

Otherwise, I am satisfied with the revisions and approve the resubmission.

Is the work clearly and accurately presented and does it cite the current literature?

Partly

If applicable, is the statistical analysis and its interpretation appropriate?

Partly

Are all the source data underlying the results available to ensure full reproducibility?

No

Is the study design appropriate and is the work technically sound?

Partly

Are the conclusions drawn adequately supported by the results?

Partly

Are sufficient details of methods and analysis provided to allow replication by others?

No

Reviewer Expertise:

deafblindness, qualitative research

I confirm that I have read this submission and believe that I have an appropriate level of expertise to confirm that it is of an acceptable scientific standard.

# Reviewer report: Reviewer response for version 2

**DOI:** 10.5256/f1000research.203922.r501104

**Published:** 2026-07-23

**Authors:** Prof. Lynne Kennedy

**Affiliations:** 1 Zayed University, Abu Dhabi, United Arab Emirates

I approve the resubmission.

Is the work clearly and accurately presented and does it cite the current literature?

Partly

If applicable, is the statistical analysis and its interpretation appropriate?

Partly

Are all the source data underlying the results available to ensure full reproducibility?

Partly

Is the study design appropriate and is the work technically sound?

Partly

Are the conclusions drawn adequately supported by the results?

Partly

Are sufficient details of methods and analysis provided to allow replication by others?

No

Reviewer Expertise:

NA

I confirm that I have read this submission and believe that I have an appropriate level of expertise to confirm that it is of an acceptable scientific standard.

# Response

**Published:** 2026-07-23

**Authors:** Dr. Richard Mottershead

**Affiliations:** College of Health Sciences, University of Sharjah, Sharjah, Sharjah, United Arab Emirates

Dear Prof. Kennedy, 

 On behalf of the research team, we would like to thank you for reviewing our article. We have assimulated your suggestions and we believe that the article is now enhanced from making the corrections. 

 kindest regards 

 Dr. Richard Mottershead

# Reviewer report: Reviewer response for version 1

**DOI:** 10.5256/f1000research.189620.r467878

**Published:** 2026-04-16

**Authors:** Peter Simcock

**Affiliations:** 1 Birmingham City University, Birmingham, UK

Thank you for inviting me to review this interesting paper, which I have enjoyed reading.  I agree with the authors that there is a paucity of research focused on siblings in the field of deafblindness, and therefore believe this paper can make a positive contribution to the field.  This is particularly the case, in my view, owing to the approach the authors took in collecting data.  However, I believe it would benefit from a number of revisions.

**Written Expression**

 Fundamentally, the paper would benefit from a rigorous edit and proof read.  There are written expression problems, awkward phrasing and grammatical errors (e.g. apostrophe use) throughout the paper, which unfortunately obscure the authors' meaning/ impact on clarity.  For example (though please note there are others):

 The sentence "The existence of people with deafblindness complexity can have emotional consequences...".  Should this be "The existence of people with the complex condition of deafblindness can result in emotional consequences…"

 The sentence "Siblings experience communication challenges, cultural gaps, kinship, and spirituality that are still challenges in Indonesia".  In what way is experiencing spirituality a challenge?  Is the intended meaning that they experience challenges in expressing their spirituality? It is not clear.

 The sentence " This study combines the 9 steps of photovoice as summarized in Table 5. ^33^ ^,^ ^42^ ^,^ ^46^ - With what is the study combining the 9 steps? With each other? This is not clear.

 The sentence “Male and female siblings reported that during the transition period, they accompanied them to maintain daily personal hygiene”.  A sentence such as “Participants reported that during the transition period, they accompanied their sibling to maintain…” would be much clearer.

 There are some incomplete sentences in the paper and unnecessary repetition, and I would suggest avoiding one sentence paragraphs, which fragment the work.  There are also typographical errors in the tables, which need addressing.

 On occasions, owing to the reference/citation system adopted, names of authors appear absent in a way that impacts on readability.  For example:

  

-
^40^ ^,^ ^41^ recommended that for the study of adolescent deafblindness, it is necessary to include siblings in different regions according to the geographical context.
- According to, ^27^ the positive and negative impacts….
- In line with ^15^ emphasized that transition is not only a matter of individuals with disabilities but also of all family members involved…
- Similar to our findings, ^53^ found that even in developed countries, siblings also serve as caregivers, in addition to other roles they must fulfil…

**General Comments**

 It becomes clear later in the paper that its focus is on the siblings of deafblind adolescents with additional intellectual impairments/learning disability.  This is stated in the research aim and the methods section, and all eight participants were the “siblings of deafblind adolescents with mental and intellectual disabilities”.  This focus should be made clear in the title of the paper and within the abstract, particularly owing to the heterogeneity of the deafblind population, critique of research that is insufficiently clear about the sub-group of deafblind people concerned, and in view of Smith’s argument about the impact failure to render this explicit has on the “just” deafblind.

 There is a lack of clarity in relation to the concept of transition in the paper, owing the differing terms that are used throughout.  These include: “family transition”; “transition”; “post-school transition”; “transition from school to home” and “deafblindness transition”.  The period, in the Indonesian context, needs to be made much clearer in the earlier sections of the paper and consistent terminology adopted.

**Abstract**

 I appreciate the context of the study, but the opening line lacks clarity. Whilst congenital deafblindness is often accompanied by intellectual impairment/mental disability, acquired deafblindness is not.  This should be rendered explicit, and reflects my comments above.

 In the abstract there is use of the hyphenated deaf-blind, which should be avoided.

**Introduction**

 Again, there is reference to deafblindness often occurring alongside multiple disabilities; it should be clear that this is congenital deafblindness.

 The phrase “Deafblindness is often considered axiomatic” does not make sense.  Reference is given to Simcock’s work. He argues that the vulnerability of deafblind people is considered axiomatic.  Is this the intended meaning here?

 Where referring to the global prevalence rates, reference should be made to severe and milder forms of deafblindness, not severe and milder forms of deafness and blindness; recognition of deafblindness as a third, discrete category of sensory impairment is essential in the field, and reflects the position adopted by the report of the WFDB which is cited.

 I suggest that the following sentence is rephrased: *Socially, the presence of individuals with deafblindness poses challenges in building interactions and becomes a burden on other family members.  *This lacks complexity and nuance, and risks reinforcing negative narratives of deafblindness, not congruent with the literature. Owing to various internal and external factors, deafblindness poses challenges for individuals and their families; it is not merely their presence or existence that results in burden.  Indeed, there are publications reporting on the positive contributions deafblind people make to their families.

 In the fourth paragraph of the introduction is the line: *Findings even reinforce the fact that individuals with deafblindness, very few live independently post-school and require family roles past this stage.  *There are written expression problems with this sentence, but it is also not clear what findings are being referred to as there is no citation.  Does this relate to the citation at the end of the sentence before? A specific study? It is not possible to determine.

 The overall structure of the introduction could be strengthened.  It tends to jump from point to point, and there is unnecessary repetition in places. For example, one paragraph starts with “Use of photovoice to research the roles and experiences of siblings living with adolescent deafblindness during the transition phase has been rarely explored” and then the following paragraph starts with “Research focusing on the voices of siblings with deafblindness during the transition period was very limited”.

 It would be helpful in the introduction to offer a brief comment on what ‘photovoice’ means, albeit described in more detail in the methods section.

**Methods**

 It is stated in this section that “Previous studies have highlighted the impact of siblings, but have been limited to specific types of disabilities and have not focused on the transition phase. It has mostly focused on the experiences of people with disabilities themselves.”.  These points are made in the introduction, and it does not seem necessary to repeat them here.

 In the ‘design’ section, there is reference to ‘the dynamics of brotherhood’.  Use of this term is unclear in view of the mixed gender of the participants.  This may be a language issue, but needs clarification.

 In the ‘settings and participants’ section, it would be useful for the reader if the term ‘family-based parenting practices’ was defined/explained.

 In the ‘data collection’ section, it is stated that participants were asked to capture photos that ‘depicted their parenting experiences’, rather than just their ‘experiences’.  Does this mean that the researchers positioned the participants as ‘parents’ before the data collection began?  And if so, what are the implications of this for the findings?

 In the ethics section, it would be helpful to render explicit that the consent secured related to the use of photos included consent to include the photos in publications.  This is otherwise a very clear and detailed section.

 The ‘data analysis’ section includes a helpful visual/ diagram.  However, use of a rather dated Braun and Clarke reference needs attention, particularly in view of their later work on Thematic Analysis.  This is evident in the authors’ suggestion that the themes ‘emerged’, which Braun and Clarke would reject.

 In the section on ‘rigor’, there is reference to ‘group discussions involving teachers, parents and social workers’, but these discussions are not described in the ‘data collection’ section.  Were these social workers and teachers involved in the focus groups? (Step 6 of the ‘Photovoice’ Process).  There is no information about these individuals as there is with other participants.

 As the authors adopt Thematic Analysis, I would have expected some content on their own positionality and the impact this had on the research and its findings.

 It is stated that “Discussions between participants and researchers reinforce dependability”, but not made clear to the reader in what way this is achieved.

 Direct quotations from the participants are in English.  Are these a translation of the original data? If so, who undertook the translation, and how was quality of translation ensured? This needs further detail, particularly as some of the quotations appear to include mistranslations. For example:

  

- *“When I brought my brother, I was often reprimanded. Because it is troublesome and noisy if I have a tantrum, I feel embarrassed and guilty…” (Participant 6).  *Should this read “…if he has a tantrum…”
- *“Even though I wasn’t invited, I always invited my deafblind brother to join the Independence Day celebrations in the village. People often looked at my brother strangely, but that was my chance to explain to them about my brother.” (Participant 6).  *Should this read, “Even though he wasn’t invited…”

**Results**

 As a qualitative study, I would suggest the subtitle ‘Findings’ rather than ‘Results’, thought this may be a formatting of the journal.  There is some very interesting data presented here.

 With the direct quotations, the participant number is helpfully included.  However, elsewhere it is stated “The urban sibling admitted….”.  Which urban sibling? This is not clear.

 Theme 1 is entitled “invisible care work”.  However, apart from brief reference to this work being invisible in one participant quotations, it is not sufficiently clear to the reader why the work being undertaken is hidden or invisible.  The tasks that siblings are undertaking is described, but what makes this work invisible?

 Theme 3 is titled Urban dan Rural divide.  Should this be Urban and Rural divide? This error (i.e. dan instead of and) is also evident in the tables.

 It is stated in this theme that “ *Long queues and paperwork bureaucracy can leave teens feeling overwhelmed, deaf, blind, and prone to tantrums and destruction”. *This lacks clarity.  How does being in a queue leave someone feeling deaf, blind?

 The direct quotation to support Theme 4, subtheme ‘sibling identity’ does not work well in evidencing the theme.

 Overall, there is scope to develop the analysis, potentially reducing the number of themes and sub-themes.  For example, how does the subtheme: Uncertain future relate to the sub-theme Future and anxiety.  There appears to be overlap here.

**Discussion**

 There are multiple occurrences of awkward written expression in this section, which would also benefit from a clearer structure.

 Should the sentence “… *findings about the experiences and roles of adolescent siblings with deafblindness*…” read “… findings about the experiences and roles of siblings of adolescents with deafblindness or “…findings about the experiences and roles of siblings of deafblind adolescents”.

**Study Limitations**

 I disagree with the authors’ assertion that the study “makes a significant contribution to strengthening the role and voice of deafblind adolescents in Indonesia” as the voices of the deafblind individuals themselves are not the focus of the work, but rather their siblings.  The research is nonetheless important, but this assertion is in my view weak.

**Implications**

 In the limitations section it is stated that “ *gender, socioeconomic status, and cultural expectations are mentioned but not deeply explored to understand how these factors interact to shape sibling roles*”.  However, in the implications section it is stated that “ *This study shows how cultural, economic, and geographical factors (urban vs. rural) affect sibling engagement”. *I cannot see how both positions can be true.

 I do hope these comments are helpful.

Is the work clearly and accurately presented and does it cite the current literature?

Partly

If applicable, is the statistical analysis and its interpretation appropriate?

Not applicable

Are all the source data underlying the results available to ensure full reproducibility?

Partly

Is the study design appropriate and is the work technically sound?

Partly

Are the conclusions drawn adequately supported by the results?

Partly

Are sufficient details of methods and analysis provided to allow replication by others?

Partly

Reviewer Expertise:

deafblindness, with a particular focus on social work and deafblindness, the relationship between law and social work practice in the field of deafblindness , qualitative research involving deafblind people/ in the deafblindness field

I confirm that I have read this submission and believe that I have an appropriate level of expertise to confirm that it is of an acceptable scientific standard, however I have significant reservations, as outlined above.

# Response

**Published:** 2026-06-13

**Authors:** Dr. Richard Mottershead

**Affiliations:** College of Health Sciences, University of Sharjah, Sharjah, Sharjah, United Arab Emirates

Response to Reviewer 4 – Dr. Peter Simcock

 We sincerely thank Dr. Simcock for his thoughtful, detailed, and highly constructive review. We greatly appreciate his recognition of the importance of focusing on sibling experiences within deafblindness research and his positive assessment of the Photovoice methodology. We have carefully considered all recommendations and have substantially revised the manuscript to improve conceptual clarity, methodological transparency, analytical depth, and written expression.

**Written expression and manuscript structure**

 In response to the reviewer's comments, the manuscript has undergone comprehensive editing and proofreading. Numerous grammatical, syntactical, typographical, and stylistic issues have been corrected. Ambiguous sentences have been revised for clarity, repetitive content has been removed, paragraph structure has been strengthened, and terminology has been standardized throughout. Particular attention has been given to improving readability and ensuring accurate presentation of concepts within the deafblindness literature.

**Conceptual clarification of deafblindness and transition**

 The Introduction has been substantially expanded and revised. We now explicitly clarify that the study focuses on adolescents with **congenital deafblindness and additional intellectual and mental disabilities**, and this distinction has been strengthened throughout the manuscript, including the title, abstract, aims, methods, findings, and discussion. Additional discussion has been included regarding the heterogeneity of deafblindness, congenital and acquired forms, communication needs, co-occurring disabilities, and support requirements.

 Ambiguous terminology, including references to deafblindness as “axiomatic,” “invisible,” or “the most vulnerable,” has been clarified, revised, or removed. We have also strengthened discussion of transition by adopting more consistent terminology throughout the manuscript and clarifying that the study focuses on the post-school transition to adulthood within the Indonesian context.

**Photovoice methodology and participant focus**

 Following the reviewer's recommendation, Photovoice is now introduced and defined earlier in the manuscript, with expanded discussion of its purpose, strengths, and suitability for exploring hidden caregiving experiences. We have clarified that the focus of the study is on siblings and their experiences rather than on the deafblind adolescents themselves. Text that could imply participant siblings were positioned as parents or that the study primarily sought to represent the voices of deafblind adolescents has been revised accordingly.

**Methodological rigor, recruitment, and reflexivity**

 The Methodology section has been substantially strengthened through additional detail regarding participant recruitment, eligibility criteria, consent procedures, photographic ethics, and data collection. We have expanded discussion of qualitative rigor, including credibility, dependability, confirmability, and transferability. A dedicated section on researcher positionality and reflexivity has been added, and the Data Analysis section has been revised to align more closely with contemporary understandings of reflexive thematic analysis. We have also clarified that themes were actively developed through engagement with the data rather than passively emerging from it.

 Additional detail has been included regarding translation of participant quotations, collaborative interpretation processes, and the role of participant reflections in thematic development.

**Findings and analysis**

 The Findings section has been substantially revised and strengthened. The original thematic structure has been consolidated into four stronger themes to improve conceptual coherence and analytical depth. Greater explanation has been provided regarding invisible caregiving, communication brokerage, advocacy, stigma, resilience, and transition-related responsibilities. Ambiguous examples and quotations identified by the reviewer have been clarified or corrected, and inconsistencies in theme naming and interpretation have been addressed.

**Discussion, limitations, and implications**

 The Discussion has been reorganized and expanded to provide greater interpretation of findings and stronger engagement with contemporary deafblindness, disability, and family research literature. The revised manuscript now incorporates Family Systems Theory, Ecological Systems Theory, and Family Resilience Theory to strengthen interpretation of sibling experiences.

 We have also revised the Limitations and Implications sections to ensure consistency and avoid overstatement. In particular, claims regarding the study strengthening the voice of deafblind adolescents have been revised to more accurately reflect the focus on sibling perspectives. Statements regarding gender, socioeconomic status, cultural expectations, and geographical context have also been refined to ensure alignment with the scope of the findings.

 We are extremely grateful for Dr. Simcock’s 'expert' review. His comments have significantly strengthened the conceptual precision, methodological rigor, analytical depth, and overall quality of the manuscript. We apologize for the length of time to complete these changes, but the modifications were substantial to meet all four reviewers' suggestions. 

 kindest regards 

 Dr. Richard Mottershead

# Reviewer report: Reviewer response for version 1

**DOI:** 10.5256/f1000research.189620.r467871

**Published:** 2026-04-06

**Authors:** Gro Hege Saltnes Urdal

**Affiliations:** 1 Western Norway University of Applied Sciences, Bergen,, Norway

Thank you for the opportunity to review this article. The study is both interesting and relevant, offering valuable insight into the situation of siblings of young adults who are deafblind and in a transitional phase. This is a perspective that is often overlooked in research, where the focus tends to be directed toward the individuals themselves or their parents. The authors’ effort to highlight the experiences of siblings therefore represents an important and original contribution.

 The method employed, Photo-Voice, is creative, innovative, and well suited to engaging participants, thereby providing access to rich and relevant experiential data. Through both the photographs and the interviews with the siblings, the study reveals challenges that are culturally and socially embedded, illustrating their daily burdens and caregiving roles.

 At the same time, there are several elements that would benefit from clearer explanation or more precise presentation in order to strengthen the article, enhance the argumentation, and make it more robust for indexing.

**Major Comments**

**Introduction**

 The introduction would be strengthened by a clearer and more comprehensive contextualization. The authors could elaborate on how deafblindness often co-occurs with additional intellectual or psychosocial disabilities, how both congenital and acquired forms affect functioning, and why deafblindness may require extensive lifelong support, including the types of communication strategies that are necessary. The claim that deafblindness is “invisible” requires clarification, and the authors should define how rural and urban areas are understood within the Indonesian context. Certain statements should also be moderated, such as describing deafblindness as “the most vulnerable” group. Furthermore, the types of burdens experienced by families, and the specific role of the transition to adulthood, should be clarified and supported with relevant literature. A justification for why the research question is restricted to siblings of young adults with deafblindness who also have mental and intellectual barriers would also strengthen the rationale.

 There are also inconsistencies, such as the claim that previous research is limited in addressing the transition period (suggesting an absence of research), while simultaneously stating that research on siblings of deafblind individuals during this phase is “very limited,” which appears contradictory.

**Method**

 The use of Photo-Voice is a creative methodological choice. However, the method is introduced only late in the Methods section while being referenced in the introduction without explanation. A brief description of the approach, including its purpose, typical applications, and why it is appropriate for this study, would improve clarity.

 A key issue for readers concerns the study sample. The sample is small and further subdivided into rural and urban families and different family constellations. The authors should therefore clarify how participants were identified and recruited, the size of the overall recruitment pool, and the ethical considerations underlying the chosen sample size. A more detailed justification is needed to demonstrate why such a small dataset is methodologically defensible, and whether the depth of the data compensates for the limited breadth. It is also important to explain how methodological rigor was ensured—particularly given the small sample and the importance of dependability and confirmability in qualitative research.

**Findings**

 The Findings section would benefit from greater depth. The themes and subthemes are relatively brief and appear superficial or insufficiently grounded in the empirical material. It is unclear whether this stems from the sample size, the analytic process, or the presentation of results. The authors could strengthen transparency and reliability by elaborating on the coding process and illustrating more explicitly how a theme emerged from the data (give an example). In addition, some inconsistencies in the thematic structure should be addressed, such as when the same activity (e.g., tidying) is categorized under both “hidden worker” and “caregiver role.” It should also be clarified how participants grouped the photographs and whether their groupings align with the researchers’ analyses.

 Several relevant variables, such as gender, socioeconomic status, and cultural expectations, are mentioned but not systematically integrated into the interpretation of the data. Given that the study highlights the absence of geographically diverse research, it is particularly important to demonstrate how such factors shape sibling roles and caregiving responsibilities. Similar clarification is needed regarding the ages and genders of the siblings.

**Discussion**

 The Discussion section would be considerably strengthened by clearer structure and greater analytical depth. The authors should engage more directly with the interpretation of their findings rather than primarily reiterating results.

**Overall**, the article represents an important contribution, but it would be further strengthened by more detailed explanations of methodological choices, clearer conceptual definitions, and more robust argumentation based on the empirical material.

 Minor comments:

- Several linguistic and structural aspects of the manuscript could be improved to enhance clarity and overall quality.
- The use of the term *deafblindness* should be consistent throughout the article.
- There are instances of uneven syntax, minor errors, and missing punctuation that should be corrected.
- In the introduction, a number of formulations require further clarification and elaboration. For example, please explain what is meant by the statement that deafblindness is often considered *axiomatic*.
- It would also strengthen the text to highlight that individuals with deafblindness constitute a highly heterogeneous group and to acknowledge the diversity within this population, for instance regarding communication methods.
- In the fifth paragraph beginning with “Although it has begun to shift,” the authors compare developing countries with countries such as Indonesia, as well as differences between cities and rural areas within Indonesia. However, the nature of these differences is not further explained.
- In the Methods section, the following sentence appears: “Previous studies have highlighted the impact of siblings but have been limited to specific types of disabilities and have not focused on the transition phase. It has mostly focused on the experiences of people with disabilities themselves.” This has already been stated in the introduction and does not need to be repeated.
- It is also unclear why the following is included when the focus of the study is on siblings: “This method was chosen to strengthen the perspective of adolescents with deafblindness who live in different geographic areas and are often overlooked in disability research.”
- Some themes are described very briefly and are not discussed in sufficient depth. These should be expanded to establish clearer reasoning and provide a more robust analytical foundation.

Is the work clearly and accurately presented and does it cite the current literature?

Partly

If applicable, is the statistical analysis and its interpretation appropriate?

Partly

Are all the source data underlying the results available to ensure full reproducibility?

No

Is the study design appropriate and is the work technically sound?

Partly

Are the conclusions drawn adequately supported by the results?

Partly

Are sufficient details of methods and analysis provided to allow replication by others?

No

Reviewer Expertise:

Deafblindness, interpreting, qualitative research, participatory research

I confirm that I have read this submission and believe that I have an appropriate level of expertise to confirm that it is of an acceptable scientific standard, however I have significant reservations, as outlined above.

# Response

**Published:** 2026-06-13

**Authors:** Dr. Richard Mottershead

**Affiliations:** College of Health Sciences, University of Sharjah, Sharjah, Sharjah, United Arab Emirates

Response to Reviewer 3 – Dr. Gro Hege Saltnes Urdal

 We sincerely thank Dr. Saltnes Urdal for her thoughtful and constructive review. We appreciate her recognition of the originality and relevance of the study, particularly the focus on siblings, the use of Photovoice, and the contribution of visual and narrative data to understanding culturally and socially situated caregiving experiences. We have carefully addressed the recommendations and substantially revised the manuscript. We apologise for the length of time to make these substantial changes. 

**Introduction and conceptual clarification**

 The Introduction has been extensively revised to provide clearer contextualisation of deafblindness, including its heterogeneous nature, congenital and acquired forms, co-occurring intellectual, developmental, neurological, and psychosocial disabilities, communication needs, and implications for independence and lifelong support. Ambiguous or overly broad terms, including “axiomatic,” “invisible,” and “the most vulnerable,” have been removed or moderated. We have also clarified how rural and urban contexts are understood within Indonesia and expanded discussion of family burden, transition to adulthood, sibling caregiving, and the rationale for focusing on adolescents with congenital deafblindness and additional intellectual and mental disabilities.

 The research gap has been clarified to avoid contradictory phrasing. We now distinguish between existing research on transition in disability and deafblindness more generally, and the much more limited research specifically addressing sibling experiences during post-school transition.

**Photovoice and methodological justification**

 We have introduced Photovoice earlier in the manuscript and expanded the methodological justification for its use. The revised text explains its participatory purpose, relevance to disability research, and suitability for exploring hidden, sensitive, and culturally embedded caregiving experiences. The Methodology section has also been restructured and expanded to provide clearer detail regarding recruitment, eligibility criteria, consent and assent, data collection, photographic ethics, and data analysis.

**Sample size, recruitment, and rigour**

 We agree that the sample size required clearer justification. The revised manuscript now explains the purposive sampling strategy, recruitment through special education schools, gatekeeper involvement, and participant eligibility criteria. Sample adequacy is justified using the concept of information power, recognising the depth of data generated through photographs, interviews, focus groups, and collaborative reflection. The Trustworthiness section has been substantially expanded to describe credibility, dependability, confirmability, and transferability, including triangulation, audit trail, reflexive memo writing, and collaborative analysis.

**Findings and thematic structure**

 The Findings section has been substantially revised. The original six themes have been consolidated into four stronger, more conceptually coherent themes to avoid superficial breadth and improve analytical depth. The revised findings provide richer interpretation of sibling caregiving, emotional burden, geography, stigma, communication brokerage, and advocacy. We have also clarified the integration of visual and narrative data and strengthened the explanation of how participant reflections and collaborative interpretation contributed to theme development.

**Discussion and theoretical interpretation**

 The Discussion has been reorganised using clearer subheadings and expanded to provide deeper analytical interpretation rather than restating results. The revised manuscript now incorporates Family Systems Theory, Ecological Systems Theory, and Family Resilience Theory to strengthen interpretation of sibling roles, transition processes, geographical inequalities, resilience, and advocacy.

**Language, structure, and consistency**

 We have reviewed the manuscript for terminology, grammar, punctuation, and syntax. The term “deafblindness” is now used consistently throughout. Repetitive wording from the Introduction and Methods has been removed, unclear sentences have been revised, and the manuscript has been edited for clarity and coherence.

 We are grateful for Dr. Saltnes Urdal’s detailed recommendations. Her feedback has significantly strengthened the conceptual clarity, methodological transparency, analytical depth, and overall quality of the manuscript.

# Reviewer report: Reviewer response for version 1

**DOI:** 10.5256/f1000research.189620.r467872

**Published:** 2026-04-01

**Authors:** Shirley Dumassais

**Affiliations:** 1 University of Montreal, Montreal, Québec, Canada

*I would like to thank the authors for the opportunity to review this paper. I found it to be an interesting and innovative study, and I truly appreciated reading it. It is indeed refreshing to see siblings included in research on lived experiences, as this perspective is often overlooked in favour of focusing on the individuals themselves or their parents. In that sense, this work makes a valuable and original contribution.*

*I also really appreciated the creative approach used to better understand the experiences of siblings of people living with disabilities with the use of Photo-Voice. The methodology is innovative, novel, and very engaging, and I commend the authors for that initiative.*

*At the same time, I do feel that the manuscript would benefit from further clarification, precision, and refinement in several areas in order to strengthen the work for indexing. My detailed comments are included below.*

**2.1. Major Comments:**

**Title & Abstract:**

**Introduction:**

**- **Please clarify how deafblindness co-occurs with other disabilities. Providing examples of etiologies associated with multiple disabilities, as well as the types of co-occurring conditions, would strengthen this point. Including examples of both congenital and acquired causes of deafblindness would also be helpful.

**- **Please provide a citation for the definition of deafblindness.

**- **Please expand on how deafblindness affects independence and explain why it may lead to a need for lifelong support.

**- **Please clarify what is meant by the statement that deafblindness is “axiomatic.”

**- **Please reconsider the statement that individuals with deafblindness are “the most vulnerable,” as this may unintentionally minimize other groups. Referring to deafblindness as “a vulnerable group” may be more appropriate.

**- **Please clarify what is meant by deafblindness being “invisible” to the general public.

**- **Please specify the types of burden experienced by families (e.g., financial, emotional, relational, educational) and provide appropriate references.

**- **Please explain why the transition period is particularly challenging, supported by relevant references.

**- **Please provide examples of strong social service supports in developed countries, as well as factors contributing to their limited availability in developing countries.

**- **Please expand the introduction by including more background literature on transition, particularly in the context of deafblindness, as this is central to the study.

 - Please also ensure consistency in terminology, as the introduction refers to “deafblindness transition” while the research objective refers to “family transition.” A clear definition of the chosen term would be helpful.

 - Please define “photovoice” when it is first introduced in the introduction and provide additional background information on this approach.

**Methods:**

 - It remains unclear whether the siblings themselves have disabilities, as the manuscript mainly draws on literature describing photovoice used with individuals with disabilities. Please clarify this point throughout the text to avoid any ambiguity.

 - The methodology section would benefit from being structured more closely in line with APA guidelines, with a clearer and more detailed description of the procedure from recruitment through to data analysis (see Purdue OWL guidelines for reference).

 - You report the ages of the deafblind individuals; however, the rationale for including this information is not clearly explained. If the findings differ based on characteristics of the deafblind person, please report these differences in the Results section.

**Results:**

 -In Tables 2–4, participant gender is reported using sex-based terminology (male/female). Please clarify whether sex or gender data were collected and ensure consistent and appropriate terminology throughout. Please consider reporting any meaningful differences in the findings based on these variables.

 - Please provide a clear rationale for the small sample size, particularly in relation to the inclusion of participants from two sibling groups (rural and urban contexts).

 - Please confirm and explain whether and how data saturation was reached.

 - The findings related to themes and subthemes appear somewhat brief and may not fully convey in-depth or novel insights. Please clarify whether this is due to the sample size. If not, consider expanding the results to better illustrate how the themes emerged.

**Discussion:**

 -The discussion section would benefit from reorganization, including the use of clear headings to improve structure and readability, as it currently feels somewhat scattered. It would also be helpful to include more interpretation of the findings, rather than mainly restating the results. In addition, I believe that if the authors address the various suggestions provided by reviewers, the discussion section will change significantly and this will ultimately strengthen the manuscript.

**2.2. Minor Comments:**

 - Please make the use of “deafblindness” vs. “deaf-blindness” consistent throughout and briefly explain the choice.

 - Please check the overall syntax, as there seem to be some inconsistencies and a few missing periods at the end of sentences.

**Title & Abstract:**

**- **The title could be made more scientific and highlight the central focus of the research which is on transition. Please consider revising accordingly.

**Introduction:**

 - The phrase “especially deafblind teenagers who are facing a transition period” appears to be an incomplete sentence. Sentence should be revised to include a verb for readability.

 - The sentence “However, in the context of developing countries, the experience of siblings with multiple disabilities, especially the deafblind, can differ significantly” should be clarified. As currently written, it may suggest that the siblings themselves have multiple disabilities.

 - The statement indicating that the experience “can differ significantly” would be strengthened by specifying the point of comparison (e.g., in relation to other contexts or populations).

 - Headings for the research objective and research question should be singular.

**Methods:**

**- **Please harmonize the wording within the tables (e.g., capitalization), and correct minor inconsistencies (e.g., “divorced” is missing a “d”).

 - Please specify how middle-, lower-, and higher-SES categories are defined, and clarify how this information was obtained.

 - Please begin the Methods section with a statement on ethical considerations.

Is the work clearly and accurately presented and does it cite the current literature?

Partly

If applicable, is the statistical analysis and its interpretation appropriate?

Partly

Are all the source data underlying the results available to ensure full reproducibility?

No

Is the study design appropriate and is the work technically sound?

Partly

Are the conclusions drawn adequately supported by the results?

Partly

Are sufficient details of methods and analysis provided to allow replication by others?

No

Reviewer Expertise:

deafblindness, qualitative research

I confirm that I have read this submission and believe that I have an appropriate level of expertise to confirm that it is of an acceptable scientific standard, however I have significant reservations, as outlined above.

# Response

**Published:** 2026-06-13

**Authors:** Dr. Richard Mottershead

**Affiliations:** College of Health Sciences, University of Sharjah, Sharjah, Sharjah, United Arab Emirates

Response to Reviewer 2 – Dr. Shirley Dumassais

 We sincerely thank Dr. Dumassais for her thoughtful, constructive, and encouraging review. We greatly appreciate her recognition of the originality of the study, the inclusion of sibling perspectives, and the innovative use of Photovoice methodology. We have carefully considered all recommendations and have substantially revised the manuscript to improve conceptual clarity, methodological transparency, scholarly depth, and overall readability.

**Title, Abstract, and Introduction**

 In response to the reviewer's comments, the title, abstract, and introduction have been revised and expanded. Additional literature has been incorporated to provide a more comprehensive discussion of congenital deafblindness, including definitions, etiologies, congenital and acquired causes, and the frequent co-occurrence of intellectual, developmental, and psychosocial disabilities. We have clarified how deafblindness may affect independence, communication, social participation, and the need for long-term support. Ambiguous terminology, including references to deafblindness as “axiomatic,” “invisible,” or “the most vulnerable,” has been revised or removed to improve precision and avoid unintended implications.

 The introduction now includes a stronger discussion of post-school transition, transition-related challenges, family-centred care, sibling caregiving, and disability support systems within both developed and developing contexts. Additional literature has been incorporated to strengthen the conceptual foundation of the study and clarify the distinction between individual transition and family transition processes. A definition and rationale for Photovoice have also been introduced earlier in the manuscript.

**Methodology**

 The Methods section has been substantially restructured and expanded to improve transparency and alignment with qualitative research reporting standards. Additional detail has been provided regarding recruitment procedures, eligibility criteria, participant characteristics, ethical approval, parental consent and participant assent processes, data collection procedures, and data analysis. We have clarified that the participants were siblings of adolescents with congenital deafblindness and additional disabilities and were not themselves identified as having disabilities.

 In response to comments regarding sample size, we have expanded the justification for the purposive sample and adopted the concept of information power (Malterud et al., 2016) rather than relying solely on theoretical saturation. The richness of the Photovoice dataset, comprising photographs, interviews, focus groups, and collaborative reflection, is now discussed as a key justification for sample adequacy. Additional discussion of trustworthiness, reflexivity, credibility, dependability, confirmability, and transferability has also been incorporated.

**Results**

 Participant tables have been reviewed and revised for consistency and clarity. Terminology relating to sex and gender has been standardised, and table formatting has been improved. Additional explanation of participant characteristics and contextual factors has been included where appropriate. The rationale for participant grouping and sample composition has also been clarified.

 Following reviewer recommendations, the Findings section has been refined through consolidation of themes to improve coherence, conceptual depth, and analytical richness. Greater emphasis has been placed on interpretation and contextual understanding rather than descriptive reporting alone.

**Discussion**

 The Discussion has been substantially reorganised using clearer thematic subheadings to improve readability and structure. Additional interpretation has been incorporated throughout, moving beyond summary of findings to deeper engagement with existing literature and theory. Family Systems Theory, Ecological Systems Theory, and Family Resilience Theory have been integrated to strengthen interpretation of sibling caregiving experiences, transition processes, resilience, advocacy, stigma, and support systems.

**Minor Revisions**

 We have reviewed terminology throughout the manuscript to ensure consistency in the use of “deafblindness.” Grammatical, typographical, formatting, and syntax issues identified by the reviewer have been corrected. Research Objective and Research Question headings have been revised to singular form, table presentation has been standardized, and additional contextual clarification has been provided where requested.

 We are grateful for Dr. Dumassais’ insightful recommendations. Her comments have significantly strengthened the clarity, methodological rigour, theoretical grounding, and overall quality of the manuscript.

# Reviewer report: Reviewer response for version 1

**DOI:** 10.5256/f1000research.189620.r454531

**Published:** 2026-02-28

**Authors:** Prof. Lynne Kennedy

**Affiliations:** 1 Zayed University, Abu Dhabi, United Arab Emirates

Thank you for the opportunity to review this manuscript, about such an interesting study.

 The article outlines qualitative findings and images from a small (n=8) study on sibling caregiving to deafblind children living in rural (n=4) and urban (n=4) areas in Indonesia. It uses the participatory action research method, Photo-Voice, to collect sensitive data, through photographs, and then interviewing sibling caregivers to understand their caregiving experiences. The findings highlight a number of themes that emerged, with respect to the culturally and socially situated challenges of the participants, as young adolescent, sibling, caregivers. 

 The study has considerable potential to illuminate the challenges and daily struggles of adolescent in Indonesia who provide caregiving to deafblind siblings, and similarly rare but challenging disabilities.  The methodology used and the data produced are novel and illuminating. Further consideration of the method used in this particular context would be helpful addition to the paper. How this method  adds value.  How appropriate it may be in helping to examine caregiving experiences associated with such socially and culturally sensitive and rare health issues. 

 A key question many readers of this manuscript are likely to raise is the study sample.   As a relatively small study - and further sub-sampled for nuclear and extended family, rural and urban living, several questions - opportunities and limitations might be raised.  

 - Was the sample size purposively small?  and why? How were participants identified or recruited. How big is the overall sampling pool. Factors influencing decisions on sampling and recruitment strategies. How the small numbers have influenced the methodology, the results/findings?  

 - Clear justification and detailed discussion of the study sample size is needed, as this will be foremost in readers minds; 

 -  Number of participants and small scale study, can this be justified by the depth of data? richness?

 - was theoretical saturation reached? on all sub groups, themes etc., 

 - The themes and subthemes are a little thin and in danger of reading superficial, or mis aligned assumptions - carefully reflect on the number and depth of themes presented; is this supported in the manuscript by the data presented? If word limit is an issue - then focus in depth on fewer priority themes, and demonstrate the rigor and quality of data, rather than trying to provide a scant overview of all photographs, and themes in the data.  

 - Temper the discussion and the conclusion to correspond with fewer themes,  illustrating richness and conceptual depth rather than breadth. 

   - You specifically refer to rigor of the study, this part is also very important - and needs expanding with clear examples of how dependability confirmability were addressed in this study - because the sample size and sub-sampling are relatively small, even for a qualitative study.  

**Further comments and recommended amendments:**

**- structure and style:** apply the PEEL approach to structuring your writing to improve the flow of the manuscript, ensures that each point made has a clear explanation, depth, through evidence and links. 

 - Avoid paragraphs with only 2 sentences. Each parag should contain several sentences. Address one key point, before linking to the next. This applies throughout, and especially in the discussion. 

 - Provide more details and define caregiving, family, caregiving in the introduction.

 You mention that 0.2% are affected globally, while important, perhaps rephrase, as it is relatively rare condition, but nonetheless, is extremely challenging condition. Is the rate higher in Indonesia? Provide more context. 

 - Introduction - why is kinship and spirituality mentioned alongside challenges - address. 

 - SHOWED framework - citation required. 

 - check for typos ( urban dan ...) throughout 

 - clarify sentence : Long queues and paperwork *bureaucracy can leave teens feeling overwhelmed, deaf, blind, and prone to tantrums and destruction. How can this lead to deaf, blind ... ?*

 - Change from limitations, to include strengths and limitations ? 

 - Temper claims in conclusion that school social workers are central - please highlight strong evidence to justify this 

 - Also - Although the results cannot be generalized, this study confirms the need for multidimensional interventions - avoid 'over-reach' of the findings, temper this. 

 -Expand on reference to the methodology, and its value, in the discussion 

*- You state - An in-depth description of the participant’s context and geography is provided to support the transferability of the findings. *However I cannot see this. Please address. 

 - Enhance the appearance and details in the Tables, Combine these? 

 - Method - should this be methodology?

 - Include a justification for a qualitative - and PAR - study design. 

 - Illustrate how the steps for rigor applied in the procedures , stages, and the analysis - in the Methods section. 

 - Ethics - was approval to approach siblings received? or permission granted via parents. 

 - Details of Recruitment procedures ? 

Is the work clearly and accurately presented and does it cite the current literature?

Partly

If applicable, is the statistical analysis and its interpretation appropriate?

Partly

Are all the source data underlying the results available to ensure full reproducibility?

Partly

Is the study design appropriate and is the work technically sound?

Partly

Are the conclusions drawn adequately supported by the results?

Partly

Are sufficient details of methods and analysis provided to allow replication by others?

No

Reviewer Expertise:

Qualitative research, participatory research, family caregiving, public health, nutrition prevention, health promotion.

I confirm that I have read this submission and believe that I have an appropriate level of expertise to confirm that it is of an acceptable scientific standard, however I have significant reservations, as outlined above.

# Response

**Published:** 2026-06-13

**Authors:** Dr. Richard Mottershead

**Affiliations:** College of Health Sciences, University of Sharjah, Sharjah, Sharjah, United Arab Emirates

Response to Reviewer 1 – Professor Lynne Kennedy

 We sincerely thank Professor Kennedy for her thoughtful, detailed, and constructive review. We greatly appreciate her recognition of the novelty of the study, the value of the Photovoice methodology, and the importance of exploring sibling caregiving experiences within the context of congenital deaf blindness in Indonesia. We have carefully considered all comments and substantially revised the manuscript accordingly.

**Photovoice methodology and methodological justification**

 In response to the recommendation for greater methodological discussion, we have expanded the Methodology section to provide a stronger justification for the use of Photovoice as a participatory action research approach. Additional discussion has been included regarding the suitability of Photovoice for exploring sensitive, culturally situated, and often hidden caregiving experiences. We have also expanded discussion of the value of visual methods within disability research and revisited this contribution within the Discussion section.

**Sample size, recruitment, and qualitative rigor**

 We agree that the small sample size required clearer justification. The Participants and Recruitment section have been substantially revised to provide additional detail regarding purposive sampling, recruitment procedures, eligibility criteria, and the rationale for participant selection. Rather than relying on notions of theoretical saturation, we have adopted the concept of information power (Malterud et al., 2016) to justify sample adequacy, recognizing the depth and richness of data generated through photographs, interviews, focus groups, and collaborative reflection. We have also expanded the Trustworthiness section to provide clearer explanations of credibility, dependability, confirmability, and transferability, including triangulation, reflexive memo writing, audit trails, and collaborative analysis procedures

**Themes, findings, discussion, and conclusions**

 Following the reviewer's recommendation, the Findings section has been refined through consolidation of themes to improve conceptual depth and coherence. The Discussion and Conclusion sections have been revised and strengthened to provide deeper interpretation of findings while avoiding over-extension of claims. Statements regarding transferability, intervention development, and professional roles have been tempered to ensure alignment with the qualitative nature and scope of the study.

**Introduction and conceptual framing**

 The Introduction has been substantially expanded. Additional discussion has been included regarding caregiving, family-centered care, sibling caregiving, deafblindness, and post-school transition. Greater contextualization of congenital deafblindness and disability support within Indonesia has been incorporated, alongside clarification of references to kinship structures, spirituality, and family relationships. The research gap and rationale have also been strengthened.

**Methodological transparency and ethics**

 Additional detail has been added regarding the qualitative and participatory research design, recruitment procedures, parental consent and participant assent processes, ethical approval, and photographic ethics. Citations have been added where required, including for the SHOWeD framework.

**Presentation, structure, and language**

 The manuscript has undergone substantial restructuring and editing to improve flow, coherence, and readability. Paragraph structure has been revised throughout to improve logical progression of ideas, and language has been refined to address typographical, grammatical, and stylistic issues identified by the reviewer. Tables have been reviewed and enhanced, and additional contextual information regarding participants and setting has been included to strengthen transferability.

 We are grateful for Professor Kennedy's insightful recommendations, which have significantly strengthened the methodological rigor, conceptual depth, and overall quality of the manuscript.
